# Identification of Real-Life Mixtures Using Human Biomonitoring Data: A Proof of Concept Study

**DOI:** 10.3390/toxics11030204

**Published:** 2023-02-22

**Authors:** Laura Rodriguez Martin, Ilse Ottenbros, Nina Vogel, Marike Kolossa-Gehring, Phillipp Schmidt, Katarína Řiháčková, Miguel Juliá Molina, Elena Varea-Jiménez, Eva Govarts, Susana Pedraza-Diaz, Erik Lebret, Jelle Vlaanderen, Mirjam Luijten

**Affiliations:** 1 Health, Flemish Institute for Technological Research (VITO), 2400 Mol, Belgium; 2 Institute for Risk Assessment Sciences (IRAS), Utrecht University, 3584 CM Utrecht, The Netherlands; 3 Center for Sustainability, Environment and Health, National Institute for Public Health and the Environment (RIVM), 3720 BA Bilthoven, The Netherlands; 4 German Environment Agency (UBA), 14195 Berlin, Germany; 5 RECETOX, Faculty of Science, Masaryk University, 625 00 Brno, Czech Republic; 6 National Centre for Environmental Health, Instituto de Salud Carlos III, 28220 Majadahonda, Spain; 7 Centre for Health Protection, National Institute for Public Health and the Environment (RIVM), 3720 BA Bilthoven, The Netherlands

**Keywords:** chemical mixtures, human biomonitoring, network analysis, combined exposure, clustering, mixture risk assessment, HBM4EU

## Abstract

Human health risk assessment of chemical mixtures is complex due to the almost infinite number of possible combinations of chemicals to which people are exposed to on a daily basis. Human biomonitoring (HBM) approaches can provide inter alia information on the chemicals that are in our body at one point in time. Network analysis applied to such data may provide insight into real-life mixtures by visualizing chemical exposure patterns. The identification of groups of more densely correlated biomarkers, so-called “communities”, within these networks highlights which combination of substances should be considered in terms of real-life mixtures to which a population is exposed. We applied network analyses to HBM datasets from Belgium, Czech Republic, Germany, and Spain, with the aim to explore its added value for exposure and risk assessment. The datasets varied in study population, study design, and chemicals analysed. Sensitivity analysis was performed to address the influence of different approaches to standardise for creatinine content of urine. Our approach demonstrates that network analysis applied to HBM data of highly varying origin provides useful information with regards to the existence of groups of biomarkers that are densely correlated. This information is relevant for regulatory risk assessment, as well as for the design of relevant mixture exposure experiments.

## 1. Introduction

Humans are exposed to a myriad of concurrent and protracted environmental, occupational, dietary, lifestyle, and consumer product exposures. Due to the (increasingly) large number of chemicals present in the environment, exposure and risk assessment of chemical mixtures is complex and poses several challenges for scientists, risk assessors, and managers [1,2]. Increasing awareness that daily-life exposure involves exposure to an almost infinite number of different combinations of chemicals, needing a move beyond chemical-by-chemical assessments, has led to a prioritisation of chemical mixtures in policy and research.

There is no broadly accepted operational definition of mixtures. The European Commission communication on “The combination effects of chemicals—Chemical mixtures” [3] was published in response to a request from the European Parliament for the Commission to consider the extent to which the existing legislation “adequately addresses risks from exposure to multiple chemicals from different sources and pathways, and on this basis considers appropriate modifications, guidelines and assessment methods”. In the communication, mixtures are differentiated as follows: (a) intentional mixtures, i.e., manufactured formulated products that are marketed as such; (b) mixtures originating from a single source, also known as ‘unintentional mixtures’; and (c) mixtures of chemicals originating from multiple sources and through multiple pathways, also known as ‘coincidental mixtures’ [4,5].

Intentional, unintentional, and coincidental mixtures can arise from combinations of ambient environments and indoor sources, food products or contamination, consumer products, cosmetics, occupational exposures, medication and medical implants, and lifestyle. In principle, every single substance, once it enters the body, will exhibit its health effects in interaction with a person’s genetic makeup and acquired characteristics, and in concert with all other (xenobiotic) substances from previous and simultaneous exposures. These mixtures thus form a challenge to (experimental and observational) science, to mechanistic and causal assessment of risks, and to regulation of substances and general risk management policies [6,7]. In this manuscript, the term ‘mixture’ is used to describe any combination of exposure to chemical substances or of exposure biomarkers that have been measured in one or more biological matrices of a person during a single time point. These biomarkers include both the chemical substances themselves and/or their metabolites.

In the context of the European Joint Programme HBM4EU (hbm4eu.eu) on human biomonitoring (HBM), we evaluated existing HBM data using correlation network analysis to identify real-life exposure patterns to mixtures in the human body. Network analysis is a graphical method to visualise correlations between variables in a dataset. The method allows for the identification of groups of exposure biomarkers that are more densely related amongst each other than with other biomarkers. These groups are referred to as “communities”. Building on a successful network analysis exploration based on Flemish data [8], we further developed and applied network analysis to HBM datasets from Belgium, the Czech Republic, Germany, and Spain. The objective was to describe the distribution of (patterns in) biomarkers of exposure and to identify possible determinants that explain observed variation of patterns in biomarkers of exposure. For each of the four studies, results of the network analyses are shown, and findings are discussed.

## 2. Materials and Methods

In this section, we first describe data selection and preparation steps, followed by the characteristics of the four datasets, the statistical descriptives and network analyses.

### 2.1. Selection of Existing HBM Studies

With the aim to further explore the added value of network analysis, four HBM studies participating in the HBM4EU project were selected. The selection of the studies was based on data availability, as well as on availability of appropriate statistical expertise at the respective institutes.

### 2.2. Data Selection and Preparation

Harmonised data selection and preparation steps were performed with the subsequent network analyses in mind. Hence, for each of the studies, the most data-rich subset was chosen in terms of the maximum number of biomarkers measured. The data preparation steps are described in more detail in Ottenbros et al. [8]. In brief, these involve (a) checking the distribution of the variables; (b) transforming the data if needed; (c) imputing the data points below the LOD (limit of detection) or LOQ (limit of quantification); (d) correcting for outliers; (e) standardizing around zero; and (f) scaling of the data.

Concentrations of biomarkers were natural log transformed because HBM distributions are typically skewed. The network analysis makes use of the partial correlation structure. Therefore, a strategy for dealing with censored and missing data is required. Thus, an (arbitrary) cut-off at a maximum of 40% of HBM levels below LOD/LOQ was applied. Substances with more than 40% of the measured HBM values below LOD/LOQ were excluded from further analysis. For the included substances, values below LOD/LOQ were imputed based on a maximum likelihood estimation via single conditional imputation, dependent on observed values for the other biomarkers [9]. Missing values in biomarkers (completely missing, e.g., due to insufficient sample volume) and determinants were imputed by using a single imputation strategy using the R package mice (version 3.15; [10]) in R (v3.5.0 or higher [11]). Please refer to the description of the individual studies (Section 2.3) for details on which determinants this strategy was applied to. Determinants (e.g., age, sex, and smoking) were imputed first, using linear regression for continuous variables and logistic regression for the binary variables. The determinants and observed values were then used as prediction matrix for single imputation of those biomarkers that were completely missing, using linear regression.

For several substances, notably metals, different species were measured. For example, for arsenic, data for total arsenic, organic, and inorganic arsenic were available. Additionally, in some studies, the same substance was measured in urine as well as in blood, e.g., lead or cadmium. This would lead to relatively high correlations between the different biomarkers for the same substance. In terms of combined exposures to chemical substances, such correlations do not provide relevant information. Furthermore, it may also affect the partial correlations structure with other substances. Therefore, only a single biomarker was selected for inclusion in the network analysis; where possible, the biomarker that best reflects the long-term exposure of the individual was selected. Furthermore, metabolites of the large group of phthalates were not summed up to their diesters but included in their monoester concentration.

For substances measured in urine, a standardisation for creatinine content was performed to take into account the dilution level of spot or morning urine samples; the dilution level could affect the correlation structure with other substances measured in urine. For lipophilic substances measured in blood, blood lipid levels were used to standardise measured blood levels. A sensitivity analysis was performed on the German data (see Appendix A), showing the results standardised for creatinine or not.

### 2.3. Characteristics of the Four Existing HBM Datasets

#### 2.3.1. 3xG (Belgium)

The 3xG study (Health—Municipalities—Birth, translated from Gezondheid, Gemeenten, Geboorten) is a birth cohort study that monitors and promotes health of the inhabitants of three bordering rural communities (Dessel, Mol, and Retie) in Flanders, Belgium. This study focuses on the effect of the environment and lifestyle on health. This is performed by researching 301 growing children from the region and by processing the disease and mortality registers of the 3 municipalities. The aim of the 3xG study is to follow-up the health and development of growing children as a sentinel population and to study the influence of environmental exposures via biomonitoring. It is one of the initiatives in the region to positively impact the well-being and welfare of the population.

All pregnant women in the region that fulfilled the inclusion criteria and were expected to give birth between 2010 and 2015 were invited to participate. In total, 301 mother–newborn pairs were obtained. All participants signed an informed consent. Inclusion criteria were to be able to fill out a Dutch questionnaire and to live in the recruitment area [12].

All participants agreed to fill in questionnaires during pregnancy and after delivery. Socioeconomic characteristics, such as the educational level of the household members, smoking habits, information on consumption of local food, and the course of pregnancy, were collected. A urine sample was collected in the second trimester of pregnancy. Birth weight, length, and head circumference of the baby at birth were collected with consent from the mothers. A blood sample of the mother and umbilical cord blood were collected at delivery and a questionnaire was filled in by the mothers at the same time point. Since not all biomarkers were measured in the same group of participants, we selected the biomarkers that ensure a subset with enough participants. Consequently, a subset of 125 mother–child pairs were included in the network analysis. Biomarkers included in the network analysis were corrected for age (in years), body mass index (BMI), and/or smoking status of the participant. Networks were stratified by education status; low ISCED (International Standard Classification of Education) is defined by participants belonging to educational levels 0–4, and high ISCED is defined by participants belonging to educational level ≥5.

#### 2.3.2. CELSPAC—FIREexpo (Czech Republic)

The CELSPAC—FIREexpo study, conducted in the Czech Republic, aimed to determine the health risks resulting from the occupational exposure of Czech firefighters and to implement measures to minimise such risks. All participants were males between the age of 18 and 35 years and non-smokers. All participants expressed and signed their informed consent before their participation in the study. The sampling campaign took place from January 2019 to June 2020. Samples of venous blood and morning urine were collected and analysed for the presence of biomarkers. More information is publicly available on the study website (https://www.recetox.muni.cz/hear/projects/celspac-fireexpo (accessed on 27 January 2023)) and in Rihackova et al. [13]. Because of the case-control study design, the analysis of the detection frequency and the imputation of values <LOQ was carried out separately for the two population groups (firefighters and controls); therefore, the list of biomarkers used for the network analysis slightly differed between the two groups. The biomarker levels were corrected for age (in years) and BMI. Stratification for sex and smoking status was not relevant for this study (all participants were male and non-smokers), and data on education level were not collected.

#### 2.3.3. GerES V (Germany)

The German Environmental Survey for Children and Adolescents 2014–2017 (GerES V) is a population-representative cross-sectional study carried out in order to determine the exposure to pollutants of the general population in Germany and their sources. GerES V investigated children and adolescents by determining, on a representative basis, the body burden of environmental pollutants and the exposure to pollutants at home, including HBM samples with more than 80 biomarkers. The study was performed in a stratified randomly selected sample design. In GerES V, a subsample (*n* = 2294) of the 3- to 17-year-old participants of the German Health Interview and Examination Survey for Children and Adolescents (KiGGS Wave 2) by the Robert Koch Institute (RKI; Berlin, Germany) was examined [14,15]. Participants of GerES V from 167 different sampling locations in Germany were visited by a trained interviewer, conducting an interview on exposure-relevant behaviour and collecting information on the living environment with the participants and their parents or legal guardians, and collecting inter alia samples of first-morning void urine and blood. For more details on both studies, see Murawski et al. [16] and Hoffmann et al. [17].

Different biomarkers were measured in subsets of participants in the nationally representative GerES V. To have the maximum number of chemical substances while avoiding high proportions of missing data, for the current analyses, data from urinary biomarkers were used that were available for a subgroup of GerES V participants (*n* = 515, aged from 3 to 17 years old). This resulted in a set of 51 different chemicals.

Biomarkers included in the networks were corrected for the determinant’s age (in years), sex, BMI, smoking status of the participant creatinine, and education of the household (ISCED). Networks were stratified by ISCED, median age, and BMI (each only correcting for the remaining determinants). A sensitivity analysis, using different dilution adjustments of creatinine, was conducted.

#### 2.3.4. BIOAMBIENT.ES (Spain)

The BIOAMBIENT.ES study was designed as a population-based cross-sectional epidemiological study representative of the Spanish workforce, with self-administered questionnaires, medical examinations, and collection of biological samples throughout the Spanish territory [18]. The study participants were selected through a stratified sample by conglomerates to guarantee the inclusion of all the geographical areas of the territory, both sexes, and different sectors of activity (services sector and others). The study population includes subjects aged 16 or older, who were residents in Spain for at least 5 years prior to the start of the study, and who attended the occupational medical examinations during 2009. The fieldwork was conducted between March 2009 and July 2010.

Of the 1892 participants who constitute the population sample of the BIOAMBIENT.ES project, 1880 subjects provided samples with sufficient whole blood volume, while 1770 subjects provided valid morning void urine samples (defined by having creatinine levels between 0.3 and 3 g/L). The epidemiological questionnaire was designed to collect basic individual information on sociodemographic data, lifestyle, environmental conditions, and some personal characteristics. Questions about the frequency of food consumption were also included to record habitual diet, as well as about recent illnesses and the use of medications. For the purpose of the network analysis, the dataset with the highest number of substances was selected, although this reduced the number of participants, since not everyone had all substances determined.

### 2.4. Statistical Analysis

#### 2.4.1. Descriptive Analysis

The descriptive analysis of the data used for network analysis largely follows the conventions developed in HBM4EU’s Work Package on data management and analysis (HBM4EU D10.12; www.hbm4eu.eu/work-packages/deliverable-10–12-update-statistical-analysis-plan-for-the-co-funded-studies-of-wp8/ (accessed on 27 January 2023)). Central tendency and distributional measures are provided to allow an assessment of the HBM levels observed. Common scripts were used to generate the tables presenting descriptive statistics.

Descriptive statistics were calculated using R (v3.5.0 or higher [11]). The number of values and missing values, percentage below LOD and LOQ, mean, standard deviation, standard error, and geometric mean were calculated using standard R functions. Percentiles (P05 to P95) were calculated by means of the quantile function (package *stats*, version 3.6.2). Descriptive statistics were calculated on the imputed values and standardised for creatinine or blood lipids (biomarker measured in urine in the case of creatinine or measured in blood in the case of lipid standardisation for lipophilic biomarkers). Pearson correlation structures in the datasets were computed and displayed using heatmaps.

#### 2.4.2. Network Analysis

Network analyses were performed as previously described [8]. After the data selection and preparation steps, partners performed the network analysis using uniform centrally prepared scripts. Network analysis was used to describe the conditional independence between multiple variables, making use of the packages *huge* and *igraph*, using R (v3.5.0 or higher [11]) [19,20]. Within these networks, a node or dot represents a biomarker, and an edge or line between two nodes reflects the conditional dependency between these two biomarkers given all other variables. The output network presents unweighted edges, only providing information on whether the edge connecting nodes is present or absent, depending on a cut-off value (lambda).

For comparison purposes, weighted network analysis, which is more computationally demanding, was applied as well, making use of the package *EGAnet* (v1.2.3 [21]) [22,23]. The output weighted network shows the strength of the edge by thickness of the line and direction of the correlation by colour of the line (green for a positive correlation and red for a negative correlation). Both the unweighted and weighted networks were estimated using the graphical lasso (GLASSO), which involves penalised maximum likelihood estimation [24]. This method is a simple and fast algorithm for estimation of a sparse inverse covariance matrix using a lambda penalty. The GLASSO cycles through the variables, fitting a modified lasso regression to each variable in turn. Regularisation of the graph was conducted along a sequence of 10 equally spaced lambdas ranging from the maximum lambda (resulting in an empty graph) to the minimum lambda set at 10% of the maximum lambda.

For the unweighted networks, the optimal lambda selection was conducted using the stability approach to regularisation selection method (StARS), which selects the optimal lambda by variability across subsamples [25]. Variability (or instability) across subsamples is defined as the fraction of times (range: 0–0.5) that two graphs disagree on the presence of an edge, averaged over all edges in the graphs. We used the default variability threshold of 0.1.

For the weighted networks, the optimal graph from the GLASSO was selected with the EBIC tuning parameter (default of 0.5). A parametric bootstrap (1000 iterations) was used to estimate the median network structure, which was then plotted as the final result.

On both the weighted and the unweighted networks, the walktrap clustering algorithm from the *igraph* package was used, which performs random walks (using a default of 4 steps) across the network to merge nodes to so-called communities in a bottom-up manner [26,27]. Nodes were coloured according to the community they were assigned to. Edges of the unweighted networks linking different communities were coloured in red, and edges within a community were coloured in black. Biomarkers within the same community were more closely related to one another than to the other measured biomarkers in the network. To the degree possible, usage of colours is standardised within each dataset, but not across datasets, nor between unweighted and weighted network graphs.

## 3. Results

### 3.1. Descriptive Statistics for the Chemical Substances Included in the Network Analysis

Table 1 shows an overview of the descriptive statistics for the HBM datasets for those chemicals that were measured in more than one country, i.e., the biomarkers for the substances included for the network analysis, the matrix in which the biomarkers were measured, their proportions below LOD or LOQ, and percentiles and geometric mean of the biomarker concentrations. Please note that the concentrations for urinary biomarkers were standardised for creatinine. Country-specific descriptive statistics of biomarker levels as used in the network analyses are presented in Supplementary Tables S1–S4. The correlation structure between biomarkers is graphically represented in the subsequent sections by heatmaps.

#### 3.1.1. 3xG (Belgium)

The following substances and substance groups were available in a selected subsample of 125 participants: metals including cadmium (Cd), nickel (Ni), chromium (Cr), antimony (Sb), copper (Cu), thallium (Tl), and lead (Pb), total arsenic (As), hydroxy pyrene (1-PYR), trans-muconic acid (TTMA), phthalates including mono(2-ethylhexyl) phthalate (MEHP), mono(2-ethyl-5-hydroxy- hexyl) phthalate (5OH-MEHP), mono(2-ethyl-5-oxo-hexyl) phthalate (5oxo-MEHP), mono-n-butyl phthalate (MnBP), mono-benzyl phthalate (MBzP), mono-ethyl phthalate (MEP), and mono-isobutyl phthalate (MiBP), and bisphenol A (BPA total) were available in morning urine (UM) samples of the pregnant mother; musks including tonalide (AHTN) and galaxolide (HHCB) were available in the blood samples (MB) of the mother after delivery; metals (cadmium, nickel, chromium, antimony, copper, thallium, managenese, and lead) and arsenic were available in cord blood (CB) samples of the newborn; and organochlorine compounds (OCs) including polychlorinated biphenyl 138 (PCB128), polychlorinated biphenyl 153 (PCB153), polychlorinated biphenyl 180 (PCB180), dichlorodiphenyldichloroethylene (p,p’-DDE), and hexachlorobenzene (HCB), and PFASs including perfluorooctane sulfonic acid (PFOS), perfluorooctanoic acid (PFOA), and perfluorohexane sulfonic acid (PFHxS) were available in cord blood plasma (CBP) samples of the newborn.

The descriptive statistics for the biomarkers included in the network analysis are given in Supplementary Table S1. Nickel measured in the cord blood of the newborn and HCB and PFHxS measured in the cord blood plasma of the newborn show the highest percentage of values below LOD/LOQ, being 37%, 24.8%, and 20.8%, respectively. Furthermore, for nickel measured in the cord blood of the newborns, the P25 value is under the LOD (Supplementary Table S1).

**Figure 1:**
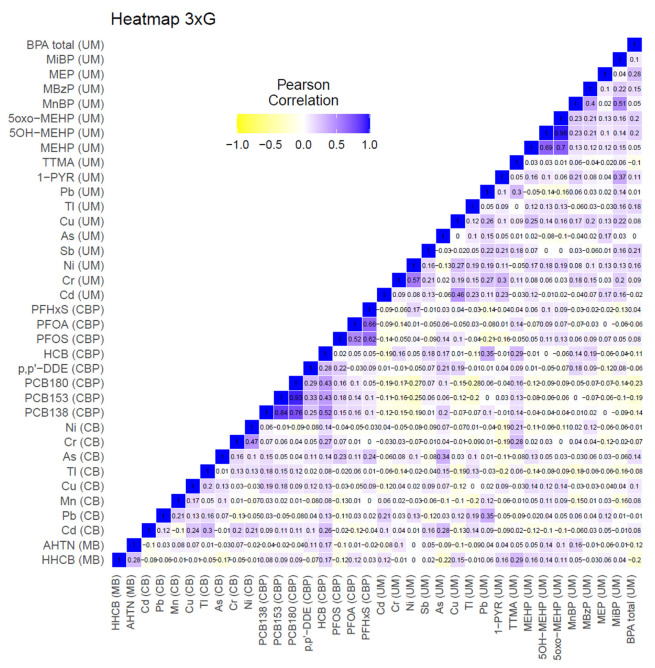
3xG: heatmap showing the Pearson correlations between all creatinine-standardised and lipid-standardised measured biomarkers measured in urine and blood, respectively, available for the selected subset of participants. Data were corrected for age, BMI, and smoking status of the participants. The matrices in which biomarkers were measured are shown between brackets (MB: maternal blood, CB: cord blood, CBP: cord blood plasma, UM: morning urine).

Figure 1 shows the correlation between the biomarkers for the abovementioned substances. Biomarkers belonging to the same chemical groups show higher correlations, such as PCBs, phthalates, PFASs, and heavy metals. Interestingly, specific heavy metals measured in the urine of the mother during pregnancy and the cord blood of the newborn during birth show low correlations. For example, arsenic and lead show a Pearson correlation of 0.34 and 0.35, respectively, while other heavy metals do not show any significant correlation. PFASs and PCBs also show small positive correlations. Significant negative correlations were not observed for any of the biomarkers.

#### 3.1.2. CELSPAC—FIREexpo (Czech Republic)

In the CELSPAC—FIREexpo study, data for the following substances were used (please note that the list of substances in the control group and firefighters might slightly differ due to differences in percentage above LOD/LOQ, see Materials and Methods): serum PFASs, i.e., PFPeA, PFHxA, PFOA, PFNA, PFDA, PFUnDA, PFBS, PFHxS, PFHpS, and PFOS, and urine OH-PAHs, i.e., 1-NAPH, 2-NAPH, 2-FLUO, 3-FLUO, ∑(2-PHEN + 3-PHEN), 1-PHEN, 4-PHEN, and 1-PYR.

Supplementary Table S2 shows the descriptive statistics for all biomarkers used in the analysis for the firefighters and the control group. The summed exposure to PFASs is significantly higher in firefighters than in the control group (Mann–Whitney U test, *p* < 0.05). When assessing individual substances, the levels of all measured PFASs are higher in firefighters than in the control group, except for PFPeA and PFUnDA. No significant difference was observed in the summed exposure levels for OH-PAHs between the firefighters and the control group (Mann–Whitney U test, *p* < 0.05); however, the levels of individual OH-PAHs slightly differ between the firefighters and the control group [13].

**Figure 2:**
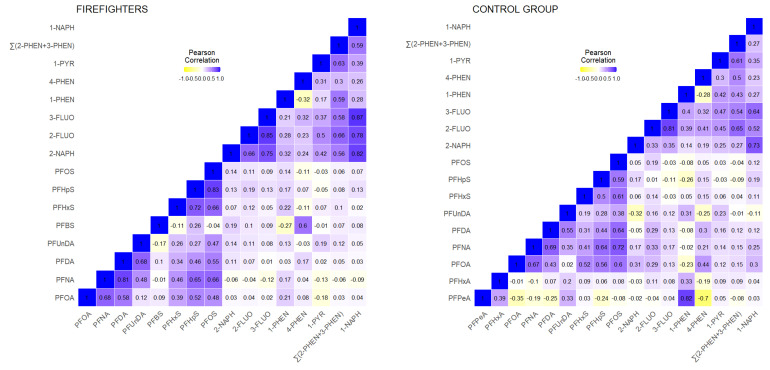
CELSPAC—FIREexpo: heatmap showing the Pearson correlations between serum PFASs and creatinine-standardised urinary OH-PAHs for firefighters (**left**) and the corresponding control group (**right**). Data were corrected for age and BMI.

Figure 2 shows the correlation heatmap for the biomarkers included for the CELSPAC—FIREexpo study. The correlations of biomarkers for substances belonging to the same family of chemicals are generally higher compared to those that belong to different chemical families. This trend is more prominent in firefighters, where the correlations within a chemical family slightly increased, while the correlations between substances from different chemical families remained weak (except for the correlation between PFBS and 4-PHEN). In the control group, the heatmap was more heterogeneous, and the within-family correlations were slightly weaker compared to firefighters, but some moderate correlations were observed for chemicals from different families.

#### 3.1.3. GerES V (Germany)

The following substances were included in first-morning void urine samples in the selected subset of 515 participants: cadmium (Cd), chromium (Cr), mercury (Hg), phthalates, DINCH, bisphenol A (BPA), polyaromatic hydrocarbons (PAHs), acrylamide, pesticides, aprotic solvents (n-ethyl-pyrrolidone; n-methyl-pyrrolidone), UV-filters (benzophenones (BP)), antimony (Sb), selenium (Se), parabens, lysmeral (TBBA), and CIT/MIT (methylchloroisothiazolinone/methylisothiazolinone). From the above set, 10 biomarkers were excluded from the network analyses because more than 40% of the measurements were below LOQ: phthalate metabolites MnOP, MnPeP, MCHP, OH-MPHP, and cx-MPHP; the aprotic solvents metabolite 5-HNEP; the pesticide glyphosate and its metabolite AMPA; and the UV-filter metabolites of BP-1 and BP-3. As a result, a total of 51 biomarkers were included in the analyses (see Table 1 and Table S3). Missing data in biomarker data were imputed as described in the Materials and Methods section (Data Selection and Preparation).

**Figure 3:**
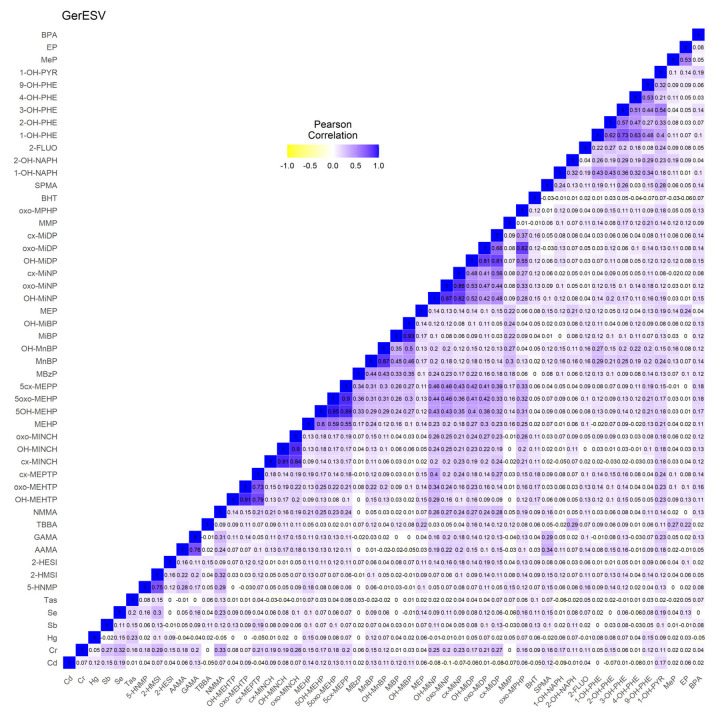
GerES V: heatmap showing the Pearson correlations between all measured creatinine-standardised biomarkers available for the selected subset of participants. Data were corrected for age, sex, body mass index (BMI), smoking status of the participant, and education of the household.

Supplementary Table S3 shows all substances included for network analyses in GerES V, their proportions below LOQ, and percentiles and geometric mean of the creatinine-standardised biomarker concentrations. Figure 3 shows the correlation heatmap for the biomarkers included for GerES V, using data standardised for creatinine and corrected for the determinants age, sex, BMI, smoking status of the participant, and education of the household. The heatmap shows mostly positive, small to medium correlations. For example, chromium and NMMA show correlations around 0.3 with several metabolites from other substance groups such as acrylamide, aprotic solvents, and some phthalates, whereas the lowest correlations with other substance groups (*r* ≈ 0–0.27) are observed for phthalate substitute DINCH, arsenic, mercury, and parabens. In contrast, correlations between metabolites of the same substance showed the highest correlations (up to *r* ≈ 0.95), e.g., acrylamide and glycidamide, and phthalates and their substitute DINCH and DEHTP.

#### 3.1.4. BIOAMBIENT.ES (Spain)

**Figure 4:**
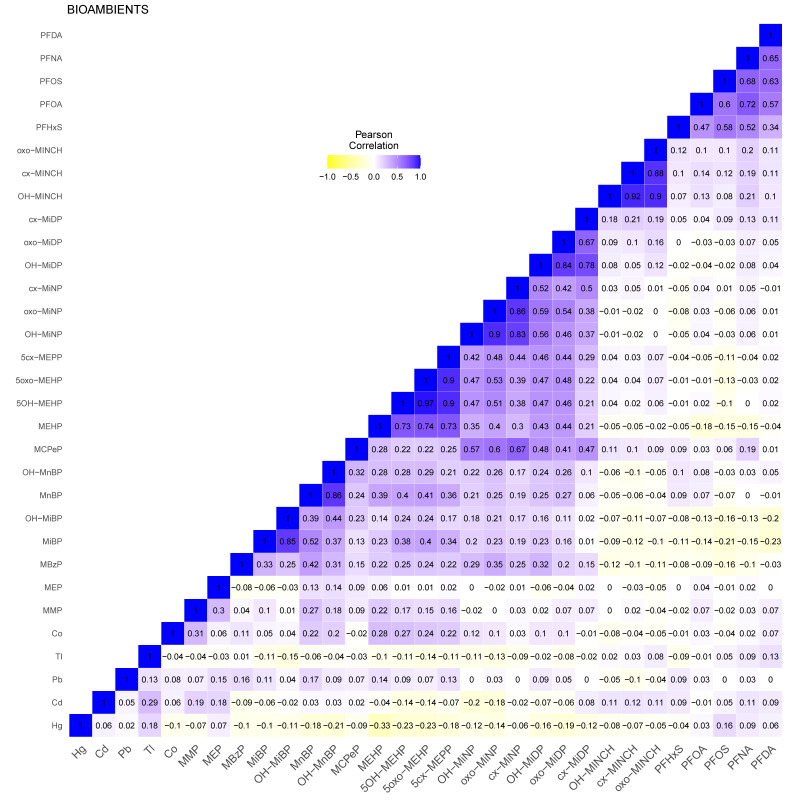
BIOAMBIENT.ES: heatmap showing the Pearson correlations between all measured creatine-standardised biomarkers available for the selected subset of participants. Data were corrected for sex, age, body mass index (BMI) and smoking status of the participants.

The selected subset of 163 participants had data on biomarkers for the following substances: metals, i.e., mercury (Hg), cadmium (Cd), lead (Pb), thallium (Tl), and cobalt (Co), phthalates (DMP, DEP, BBzP, DiBP, DnBP, DEHP, DiNP, and DiDP), DINCH, and PFASs (PFHxS, PFOA, PFOS, PFNA, and PFDA). As a result, a total of 31 biomarkers were included in the analyses. Metals and phthalates were measured in valid morning void urine and PFAS in blood. Missing values in biomarker data were imputed as described in the Materials and Methods section. Descriptive statistics for this set of biomarkers are shown in Table 1 and Table S4. Figure 4 shows the correlation heatmap for the biomarker included for BIOAMBIENT.ES, using data standardization for creatinine and corrected by age, sex, body mass index (BMI), and smoking status of the participant. The heatmap showed positive and negative, mainly small to medium correlations. The correlation among metabolites of the same group of substances showed higher positive correlations, except for metals and some phthalates such as MEP. In addition, some negative correlations were observed among PFAS or DINCH, and most of the phthalates. Mercury and thallium showed negative correlations with most biomarkers, except for PFAS.

### 3.2. Network Analysis

The network analyses produce a graphical representation of the conditional independence between the observed biomarker levels. Different colours in the networks indicate the clustering structure or communities and which biomarkers are more closely related to one another compared to the rest of the network. The sensitivity analysis of the networks consisted of two parts. The first part comprised a comparison of two weighted network estimation approaches. This was performed on the Belgium 3xG data. Secondly, the impact of different approaches on correcting biomarker levels against creatinine levels (as a measure for the level of dilution of the urine sample) was evaluated using the German GerES V data. The results of both comparisons are presented in Appendix A.

#### 3.2.1. 3xG (Belgium)

**Figure 5:**
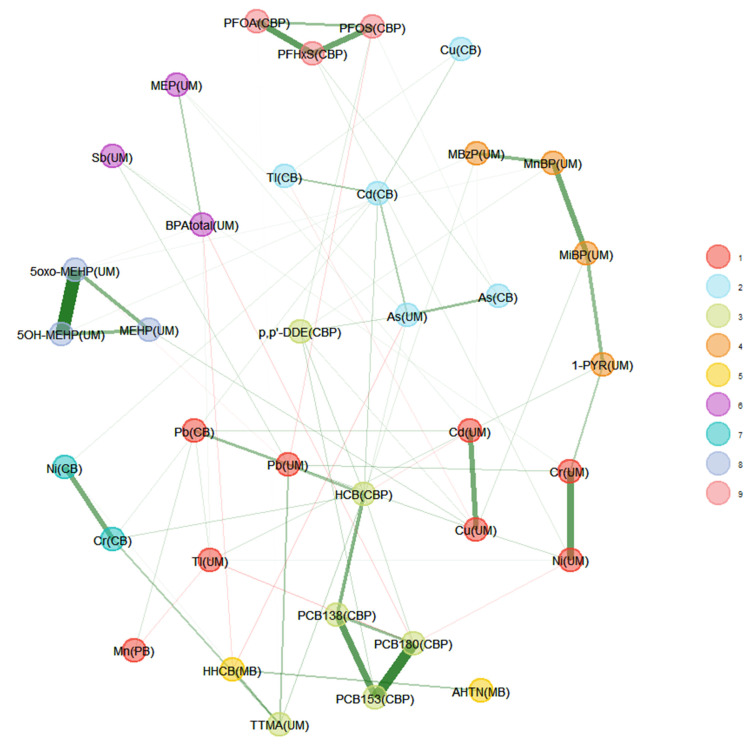
Weighted network for 3xG. The data were corrected for age, smoking, and BMI. Urinary markers were standardised for creatinine and lipid soluble blood markers were standardised for lipids. Matrices in which biomarkers are measured appear between brackets. Green lines represent a positive dependency between nodes (biomarkers) while red lines represent a negative dependency.

Figure 5 shows the weighted network for the 3xG subset of participants (*n* = 125). Biomarkers measured in urine are standardised for creatinine and lipid-soluble biomarkers in blood are standardised for lipids. Nine different communities were identified (represented by the different colours), with the strongest relations within the communities (thick lines). Negative correlations (red lines) were minimal. Green lines represent positive associations while red signify negative associations between biomarker levels. Communities with biomarkers originating from the same chemical group were detected, such as the musks (HHCB and AHTN, community 5 in yellow) or the heavy metals. The heavy metals were, however, split into three separate communities (numbers 1, 2, and 7 in Figure 5).

**Figure 6:**
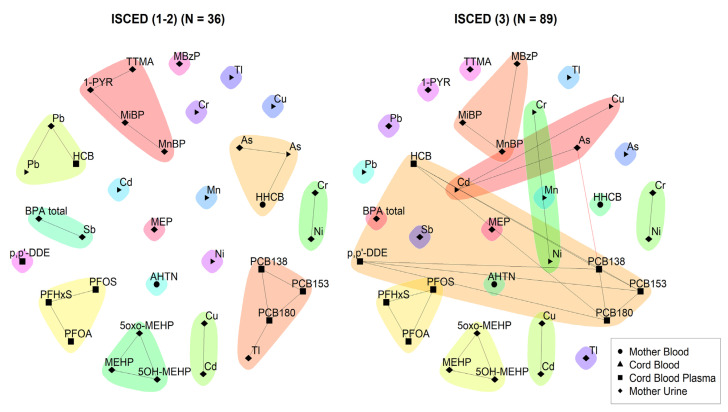
Unweighted network for 3xG for participants with low educational level (**left**) and participants with high educational level (**right**). The data were corrected for age and BMI. Urinary markers were standardised for creatinine and lipid soluble blood markers were standardised for lipids. Low ISCED is defined by participants belonging to educational levels 0–4 according to the ISCED (International Standard Classification of Education) and high ISCED is defined by participants belonging to educational level ≥5. Black lines indicate dependency between nodes (biomarkers) within a community; red lines indicate dependency between nodes in different communities.

In line with what was observed in the heatmap of 3xG (Figure 1), As and Pb measured in the urine of the mother during pregnancy and in the cord blood of the newborn at birth are highly related, which is in agreement with previous studies on the migration of hazardous heavy metals through the placenta to the foetus [28,29]. Other interesting communities can be observed in Figure 5. For example, community number 6 shows a relationship between total BPA, MEP, and Sb measured in the urine of the mother during pregnancy. Both BPA and phthalates have been found in packaging for cosmetic and personal care products and food packaging materials [30,31], and the use of make-up has been previously associated with an increase in BPA and MEP in urine [32]. The relationship between Sb and total BPA could be explained due to their presence in plastic containers that leach plasticisers and plastic additives into water or other food products [33]. Interestingly, this association is not seen in Figure 6 in a subset of participants with high educational level compared to a subset of participants with low educational level, which may be due to the fact that women with a higher educational level are more aware of the leaching of chemicals from plastic containers to water or food products. The relation of total BPA with MEP was not detected in either network once the data was stratified. Overall, the networks observed for the higher educated subset appear to be more connected with larger communities, having more (red) connections between nodes across communities.

**Figure 7:**
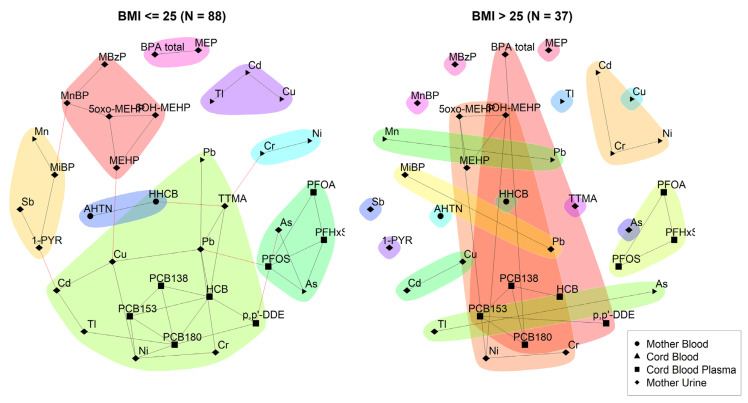
Unweighted network for 3xG for participants with a BMI ≤ 25 kg/m^2^ and participants with a BMI > 25 kg/m^2^. The data were corrected for age and smoking. Urinary markers were standardised for creatinine and blood markers were standardised for lipids. Black lines indicate dependency between nodes (biomarkers) within a community; red lines indicate dependency between nodes in different communities.

In Figure 5, another interesting community is the one consisting of 1-PYR and MiBP, MnBP, and TTMA. The most important route of exposure for 1-PYR is through smoking; however, living in a highly polluted environment also has an influence on the 1-PYR levels [34]. No common route of exposure for 1-PYR and MiBP has been found in the literature. It is intriguing to notice that the link is no longer found in the network for participants with a high level of education, but it is conserved in those with a low educational level, as seen in Figure 6. Furthermore, we also noticed that the link is no longer conserved in participants with a BMI > 25 kg/m^2^ while it is in participants with a BMI ≤ 25 kg/m^2^ (Figure 7). Figure 7 also shows more dependencies in the low BMI category, where all substances are part of a community, with some communities comprising multiple chemical families. Additionally, some dependencies across communities can be observed. Moreover, we observe again a community of BPA and MEP. In contrast, the high BMI category displays smaller communities and many substances not part of a community.

**Figure 8:**
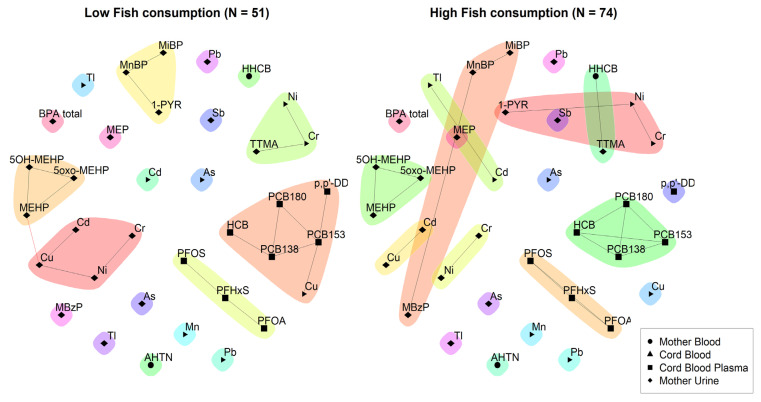
3xG: unweighted network for participants with a low fish consumption (**left**) and participants with a relatively high fish consumption (**right**). The data were corrected for age and smoking. Urinary markers were standardised for creatinine and lipid soluble blood markers were standardised for lipids. Low fish consumption is defined as consumption of fish less than 1–3 times per week and high fish consumption is defined as fish consumption of at least 1–3 times per week. Black lines indicate dependency between nodes (biomarkers) within a community; red lines indicate dependency between nodes in different communities.

Further stratifications were explored in Figure 8 where networks are explored for participants with a low fish consumption (less than 1–3 times per week) and relatively high fish consumption (equal or more than 1–3 times per week). While some communities are conserved, such as the PFASs, DEHP metabolites, and urinary heavy metals (Cu, Cd, Cr, and Ni), some others show slight changes, especially regarding other heavy metals measured in the cord blood of the newborn at birth.

#### 3.2.2. CELSPAC—FIREexpo (Czech Republic)

**Figure 9:**
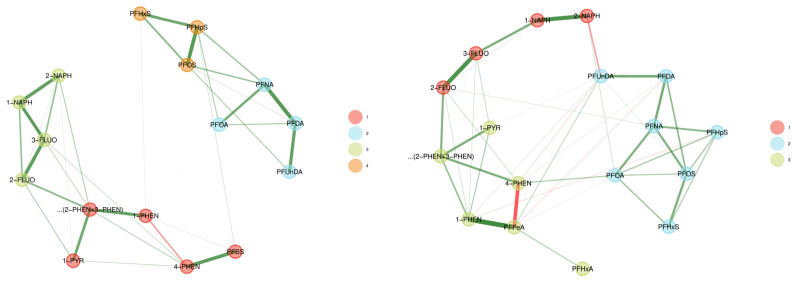
Weighted network for CELSPAC—FIREexpo firefighters (**left**) and the control group (**right**). The data were corrected for age and BMI. Urinary markers (OH-PAHs) were standardised for creatinine. Green lines represent positive associations while red signify negative associations between biomarker levels.

Figure 9 shows the weighted network of the firefighters (*n* = 52) and the control group (*n* = 55) of the CELSPAC—FIREexpo study. The set of biomarkers differ between the two groups due to differences in percentage detected above LOQ (Supplementary Table S2).

In the firefighters’ network, most PFASs and OH-PAHs clustered together in a community of the same chemical group. Two communities were created in the PFASs group (numbers 2 in blue and 4 in orange), and two in the OH-PAHs group (naphthalenes and fluorenes in community 3 in green, and other OH-PAHs in 1 in red). The exception was PFBS which was strongly linked to 4-PHEN, and therefore included in the community of OH-PAHs, rather than PFASs. In the control group network, three communities were detected: a community of naphthalenes and fluorenes (1, red), a community of seven PFAS (2, blue), and the rest of the compounds (other OH-PAHs, PFHxA, and PFPeA in the green community, 3).

In the firefighters’ network, the intra-community links were strong, and there were weak inter-community links, resulting in more strictly separated PFASs and OH-PAHs communities, while in the control group, more inter-community links were present, resulting in communities with substances from different chemical families. This might be caused by the firefighting occupation being the predominant exposure factor contributing to the PAHs and PFASs exposure in firefighters. In the controls, the levels of PFASs and PAHs are, in general, lower than in firefighters and there might not be a predominant exposure source contributing to stronger communities of PFASs and PAHs.

#### 3.2.3. GerES V (Germany)

**Figure 10:**
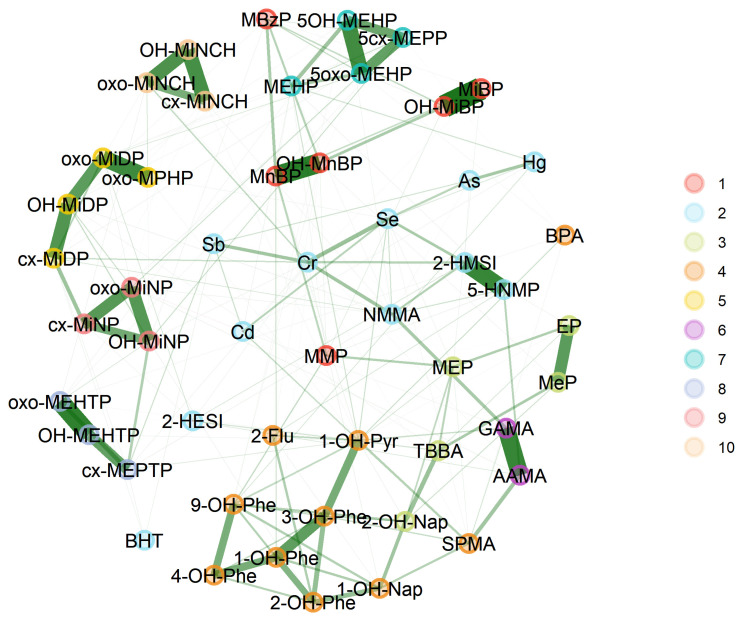
Weighted network for GerES V subsample, using creatinine-standardised and creatinine-adjusted data. Data were corrected for age and BMI. Green lines represent a positive dependency between nodes (biomarkers).

The weighted network for GerES V, allowing for assessment of the strength of the links between substances, is shown in Figure 10. Ten communities were identified. Links were stronger (i.e., thicker lines) within substance groups and among metabolites from the same parent compound; the strongest links were observed within acrylamide, aprotic solvents, parabens EP and MeP, DINCH, DEHTP, and several phthalates.

**Figure 11:**
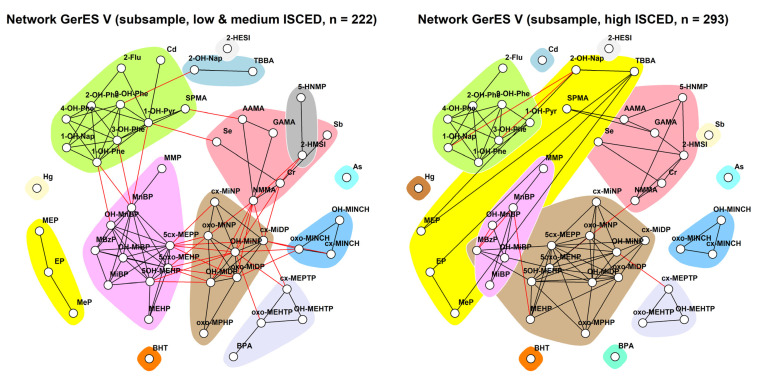
Stratification of the network for the GerES V subsample by education (ISCED), using creatinine-standardised and creatinine-adjusted data. Data were corrected for sex, smoking status, age, and BMI. Low ISCED reflects educational levels 0–4 from the ISCED (International Standard Classification of Education) and high ISCED reflects educational level ≥5. Black lines indicate dependency between nodes (biomarkers) within a community; red lines indicate dependency between nodes in different communities.

Comparison of the two networks stratified by education (Figure 11) revealed more differences than similarities. Few communities can be identified as similar between both groups, namely those of DINCH metabolites (blue), DEHTP metabolites (lavender), and PAHs (green). However, even within these communities, some remarkable differences can be observed between the groups. In the subset of participants from households with low to medium education (left panel), DEHTP co-occurs together with BPA, which is not the case for the higher educated subset. Similarly, PAHs co-occur with cadmium and benzene (SPMA) in the lower educated subset, while this co-occurrence was not observed for participants with a higher level of education. Additionally, the networks for phthalates are different between the groups, with the major difference being DEHP: this substance is part of a different community of phthalates in each education group. Furthermore, phthalate substitutes are more inter-related with communities of phthalates among participants of low to medium educated households but occur more distinctly in children and adolescents from higher educated households. In contrast to the 3xG observation, in GerES V, connections between nodes across communities are more prominent in the lower education group.

**Figure 12:**
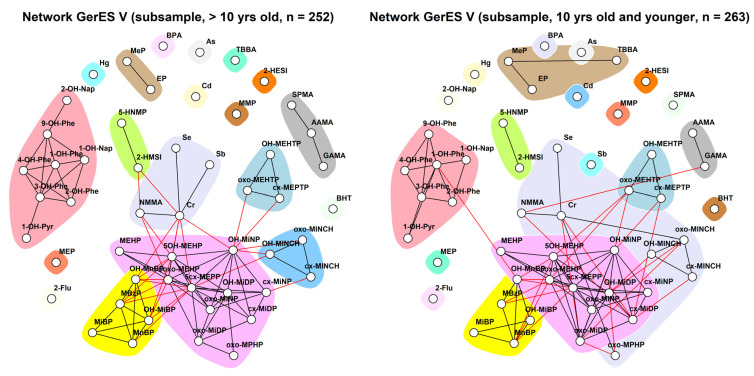
Stratification of the network for the GerES V subsample by median age (10 years old), using creatinine-standardised and creatinine-adjusted data. Data were corrected for sex, smoking status, ISCED, and BMI. Black lines indicate dependency between nodes (biomarkers) within a community; red lines indicate dependency between nodes in different communities.

Figure 12 shows stratified networks by the median age of the GerES V subset, which was 10 years. Both children older than 10 and 10 years old and younger show a community each for PAHs (light pink), two aprotic solvents (HNMP and HMSI, green), and DEHTP (light blue) metabolites. Interestingly, DINCH forms a community with NMMA and elements selenium and chromium (salmon) in younger but not older children in which each element and DINCH belong to three separate communities. In addition, the parabens—a sometimes observed standalone community—form their community with TBBA in the younger group.

**Figure 13:**
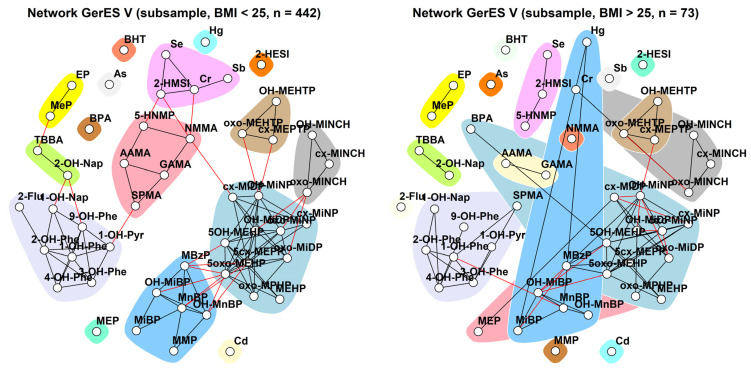
Stratification of the network for the GerES V subsample by BMI, using creatinine-standardised and creatinine-adjusted data. Data were corrected for sex, smoking status, age, and ISCED. Black lines indicate dependency between nodes (biomarkers) within a community; red lines indicate dependency between nodes in different communities.

When comparing participants with a BMI ≤ 25 lower versus participants with a BMI > 25 (Figure 13), we observed that for participants with a higher BMI (right panel), communities are more likely to include substances from other substance groups or substances which usually stand alone. For example, the PAHs community includes in addition SPMA (salmon), the phthalate community of DnBP and DiBP co-occurs with mercury (blue), the phthalate community of DiNP, DEHP, DiDP, and BBzP co-occurs with BPA, and DINCH metabolites co-occur with chromium.

#### 3.2.4. BIOAMBIENT (Spain)

**Figure 14:**
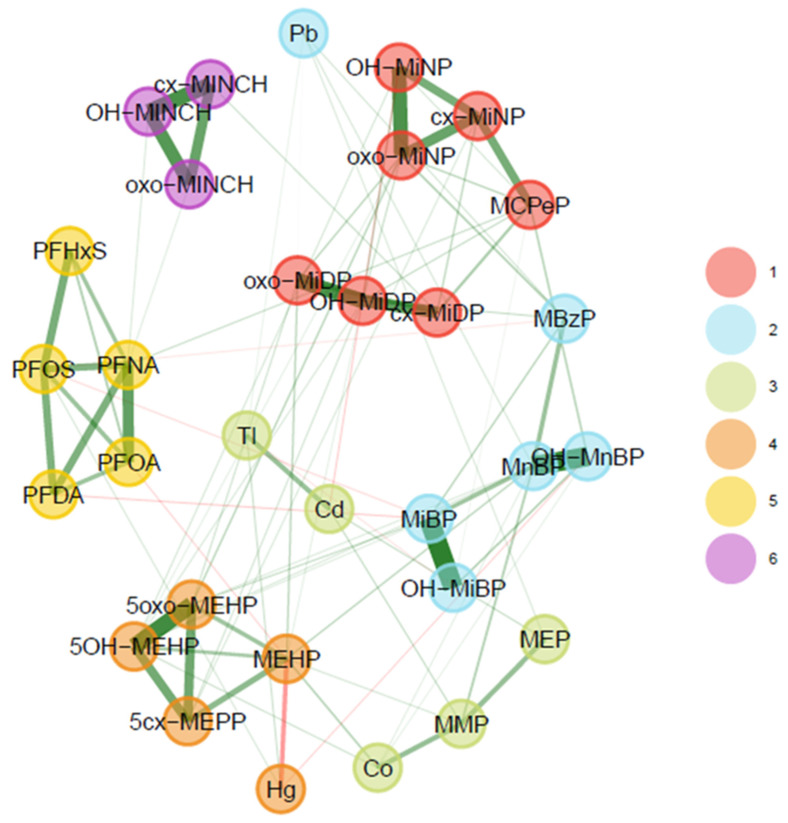
Weighted network for BIOAMBIENT.ES subsample, using urinary (creatinine-standardised) and blood data. Data were corrected for sex, smoking status, age, and BMI. Green lines represent positive associations; red lines signify negative associations between biomarker levels.

Figure 14 shows the weighted network for the BIOAMBIENT.ES dataset (*n* = 163). The graph shows six communities, with mostly positive dependencies between substances. As in the other studies, the strongest dependencies were observed in communities of substances from the same chemical family. Nonetheless, in addition to communities from the same chemical family, dependencies across chemical families were also observed. For example, several metals form communities with phthalates (community 2, 3 and 4). 

Figure 14 also shows separate grouping within parent compounds in the case of phthalates: DiBP metabolites (MiBP and OH-MiBP), DEHP metabolites (MEHP, OH-MEHP, oxo-MEHP, and cx-MEPP), DiNP metabolites (OH-MiNP, oxo-MiNP, and cx-MiNP), and DiDP metabolites (OH-MiDP, oxo-MIDP, and cx-MIDP). However, for DnBP, two metabolites (MnBP and OH-MnBP) were grouped with DiBP, whereas MCPP was grouped together with the DiNP metabolites showing strong links to cx-MiNP.

**Figure 15:**
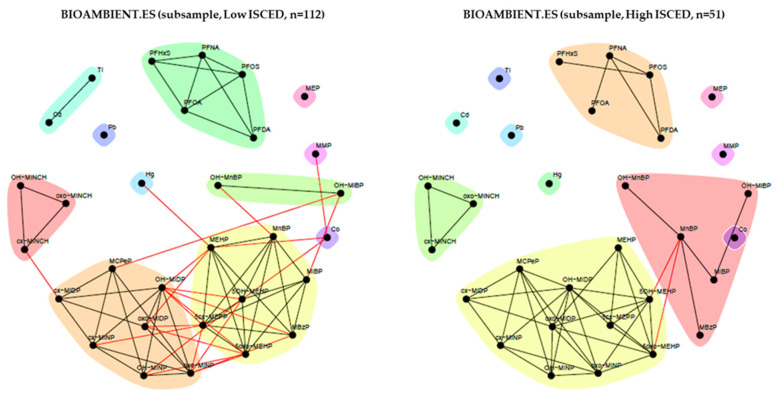
Stratification of the network for the BIOAMBIENT.ES dataset by education (ISCED level). Low ISCED (left panel) is defined by participants belonging to educational levels 0–4 according to the ISCED (International Standard Classification of Education) and high ISCED (right panel) is defined by participants belonging to educational level ≥5. Data were corrected for sex, age, BMI and smoking status. Black lines indicate dependency between nodes (biomarkers) within a community; red lines indicate dependency between nodes in different communities.

**Figure 16:**
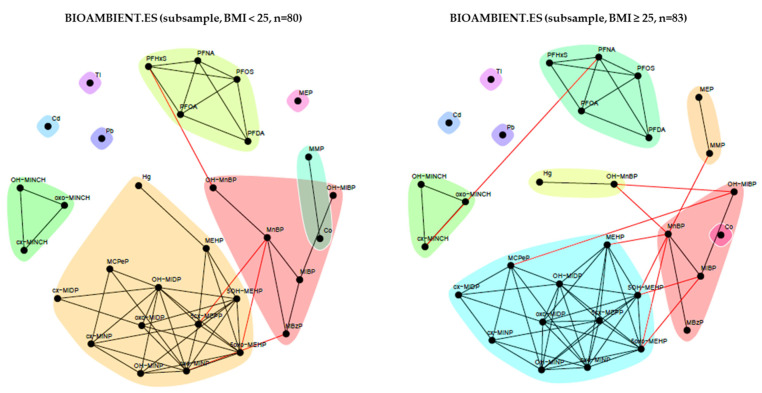
Stratification of the network for the BIOAMBIENT.ES subsample by BMI. Unweighted network for participants with a normal weight (defined as BMI < 25) is shown in the left panel, while the network for participants with overweight (BMI ≥ 25) is shown in the right panel. Data were corrected for sex, age and smoking status. Black lines indicate dependency between nodes (biomarkers) within a community; red lines indicate dependency between nodes in different communities.

**Figure 17:**
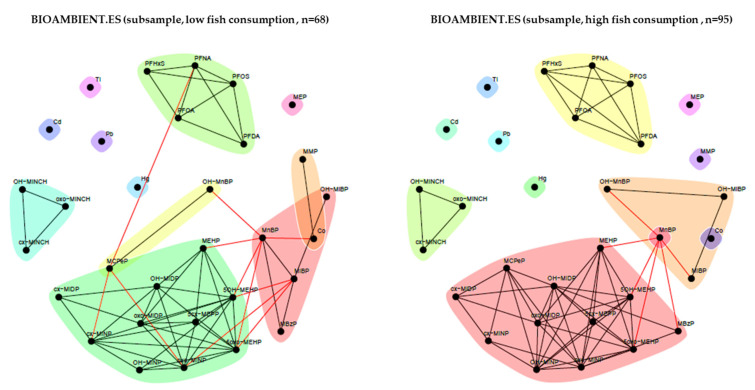
BIOAMBIENT.ES: unweighted network for participants with a low fish consumption (left) and participants with a relatively high fish consumption (right). The data were corrected for sex, age, BMI and smoking status. Low fish consumption is defined as consumption of fish less than 1–3 times per week and high fish consumption is defined as fish consumption of at least 1–3 times per week. Black lines indicate dependency between nodes (biomarkers) within a community; red lines indicate dependency between nodes in different communities.

We performed stratified unweighted network analysis for relevant determinants, including educational level (ISCED), BMI, and fish consumption. The networks identified with unweighted analysis showed fewer communities than the weighted network, possibly because of the smaller number of observations within strata (Figure 15, Figure 16 and Figure 17). Here, metals tend to appear as standalone compounds, DINCH metabolites form a distinct community, as generally so do PFAS metabolites and phthalates metabolites (two main big communities plus MEP). Communities of these two latter substance groups present some differences depending on the stratification. MEP, a metabolite of the phthalate substance DEP, often appears separate from other phthalates and substances.

Figure 15 shows the stratified networks by education level. For phthalates, in the lower ISCED level, each substance appears in a separate community, with the exception of MCPP, a metabolite of DnBP, which, as seen earlier, appears in the same community as the metabolites of DiNP. In contrast, in the high ISCED group, there are two main communities showing dependencies between them. Similar to the GerES V study, the community of DINCH metabolites was not different between the groups.

In the stratification by BMI (Figure 16), we observed that for participants with lower BMI (BMI < 25), mercury is included in DEHP, DiNP, and DiDP community, whereas in the high BMI group (BMI ≥ 25), mercury and OH-MnBP form a separate community.

When evaluating the effect of fish consumption (Figure 17), we observed communities, mainly comprising substances from a single chemical family, in both groups. In participants with a relatively low fish consumption, MCPP – OH-MnBP and MMP – Co were grouped in independent communities.

## 4. Discussion

In this study, we applied network analysis to HBM datasets from Belgium, Czech Republic, Germany, and Spain, with the aim to further explore its added value for mixture risk assessment. The network approach combined with a clustering algorithm (community detection) proved to be an intuitive graphical manner to describe the correlation structure in a dataset, taking into account all exposure markers in the mixture. Application of the network analysis in this study revealed some new insights in inter-dependencies within each dataset. Importantly, pan-European application of these methods and their interpretation would require harmonisation across Europe in terms of study design, biomarker media, chemical analysis, and the substances that are assessed. Overall, the four studies yielded diverse correlations, with more positive than negative associations (Figure 1, Figure 2, Figure 3 and Figure 4). With the exception of parent–metabolite relations, correlations were generally below 0.8, while negative correlations were generally below 0.3. It should be noted that in this study, the focus was rather on the dependencies between biomarkers (correlation structure), and not so much on the absolute levels of exposure. Nonetheless, when interpreting differences or commonalities in community patterns across studies, one should be aware that sometimes marked differences exist between studies in biomarker levels, sometimes up to one or two orders of magnitude. These may reflect differences in study population, in design, chemical analytical procedures, and actual differences in exposure patterns between study populations. In case the output should be used for prioritising mixtures of concern, of course the absolute levels should be considered as well.

The network analysis identified in all four studies, as expected, several communities of chemical families, e.g., phthalates and PAHs. Additionally, links between parent substances and metabolites were observed, e.g., for acrylamide and glycidamide. However, also exposure patterns involving substances from different chemical families were observed. Examples include the dependency between 1-PYR (biomarker for PAHs), TTMA (biomarker for benzene), and the phthalates MiBP and MnBP in the 3xG study, and the dependency between acrylamide, its metabolite glycidamide, SPMA (biomarker for benzene), and aprotic solvents (NMMA, HNMP, and HMSI) in the GerEs V study. In the CELSPAC—FIREexpo study, the network analysis revealed both positive (e.g., 4-PHEN and PFBS in firefighters) and negative (e.g., 4-PHEN and PFPeA in controls) dependencies between PAHs and PFASs. Such communities, comprising substances from different chemical families, possibly reflect a commonality in exposure patterns and thus reflect real-life mixture patterns. The communities observed may also be impacted by similarities in physicochemical properties of the substances involved. Our findings also show that in the German and Spanish data, metals (e.g., arsenic and mercury) were not always part of communities, in contrast to the Belgian data. Additionally, in the German weighted network (Figure 10), BPA was not part of a community, while a relatively strong correlation between BPA and MEP was observed in the Belgian weighted network (Figure 5). In contrast to the mostly positive links observed in the weighted networks in the three larger studies, in the smaller CELSPAC-FIREexpo control group network, a negative dependency could be observed (between 4-PHEN and PFPeA).

The unweighted network analysis stratified by covariates demonstrated differences in the community patterns. These may reflect differences in exposure patterns and pathways between strata, although no clear interpretation can be given at this point. The differences between strata may also reflect some sample differences between strata. The stratified unweighted networks also show many dependencies across communities, as indicated by the red lines in the graphs. Even though the unweighted network analysis showed differences between strata, no obvious immediate clues about sources or exposure pathways were observed. Nonetheless, the communities in the network analysis may hold some indications about relevant exposure routes. For example, the community of parabens (MeP and EP), preservatives in cosmetics, with lysmeral (TBBA), a fragrance in cosmetics, in the German network results would point at the role of cosmetics.

The above (and other) differences between studies may deserve further investigation; however, we here explicitly abstain from doing so because of the differences in study designs. Firstly, the populations sampled highly differ across the four studies. The German study focused on exposure in adolescents, the Spanish study on subjects aged 16 or over, while the Belgian study combined data from mothers and newborns, with different time points of sampling, and the Czech study focused on occupational exposure in firefighters. Additionally, the biomarkers, and thus the substances, included in the four studies vary. The same applies to the matrices in which biomarkers were determined: in the German study, only urine samples were used, while in the other three studies also blood samples were included. Hence, differences observed between the studies may stem not only from differences in exposure patterns, but also from differences in various aspects of the study designs. For a better interpretation of cross-country differences, a harmonised sample collection and laboratory analysis would be beneficial.

The analyses applied comprised both weighted and unweighted network analyses. The weighted and unweighted network analyses yielded generally similar results (data not shown). While weighted network analysis is more computationally intensive and less fit for high dimensional data in comparison to the unweighted networks, a clear advantage is the indication of the relative strength of the links and the direction of the association [35]. For a comparison between determinants within a study, only unweighted networks were used for their ease in interpretation (occurring or not-occurring edges between the biomarkers). Future work could also include a comparison between determinants based on weighted correlation networks.

The results of our study clearly show that network methods become more informative when biomarkers for a larger number of substances are included in the HBM dataset, as demonstrated, e.g., by the findings for the GerES V study versus the CELSPAC—FIREexpo study. Existing HBM studies typically have a limited number of individuals in which a wide range of chemical substances has been measured. This hampers the potential to identify patterns of chemical mixtures, and even more so to study the role of determinants, with fewer observations per stratum. For future studies, we therefore recommend to expand, where possible, the number of observations with a wide(r) range of chemicals, to improve the ability to identify real-life mixtures and to study determinants of the patterns observed.

Regarding the methodology applied, some aspects certainly deserve further improvement. Firstly, better insight into the stability and consistency of the identified networks and communities is needed [36]. Further work should also include characterisation of the uncertainty in the networks, and the decision for the community detection algorithm [26]. Better insight into aspects such as the impact of measurement errors on the networks and communities identified will enhance the appreciation of the possibilities and limitations of network analysis of HBM data for mixture risk assessment. This is crucial for its acceptance and implementation in regulatory risk assessment. Further work should also be conducted on the interpretation of the communities and the possible impact for regulatory risk assessment. We consider it crucial to take into account the toxicological properties and mechanisms of the chemical substances included in a community, because this may indicate which communities might be of more toxicological concern compared to others. Furthermore, in cases where chemicals from different families appear together in the same community, the different families may fall under different legislations and/or regulations. Such a situation would give rise to the question of how to deal with this in regulatory risk assessment.

Taken together, our study demonstrates that network analysis of HBM data allows for the identification of real-life exposure patterns to chemical mixtures occurring at a single point in time in the human body. Network analysis can be a good addition to other data explorative methods, such as heatmaps or principal component analysis. The derived networks and accompanying communities should, therefore, not replace existing methods, but rather complement and assist researchers in the description of complex mixtures in HBM data.

Graphical visualisation of the networks and communities identified greatly aids the interpretation of the output. Weighted network analysis reveals the strength and direction of the links between substances identified as co-occurring, while stratification provides insight into the impact of determinants on the exposure patterns. These features make network analysis of HBM data a useful, valuable tool for mixture risk assessment.

## Figures and Tables

**Table 1:** Descriptive statistics for biomarkers included in the network analysis measured in more than one study. PFAS were measured in blood(in µg/L), all others in urine (in µg/g creatinine).

Substance Group	Biomarker	3XG (Belgium)					CELSPAC—FIREexpo; Controls (Czech Republic)					GerES V (Germany)					BIOAMBIENT.ES (Spain)				
Distribution		% < LOQ	P25	P50	P75	P95	% < LOQ	P25	P50	P75	P95	% < LOQ	P25	P50	P75	P95	% < LOQ	P25	P50	P75	P95
**Elements**	Cd	0%	0.21	0.28	0.37	0.54						26%	< LOQ	0.06	0.09	0.15	2.5%	0.12	0.2	0.38	0.72
	Cr	1.6%	0.26	0.49	0.82	1.76						7.8%	0.26	0.34	0.49	0.77					
	Hg											5.1%	0.04	0.06	0.1	0.26	0.68%	0.56	0.99	1.58	2.75
	Sb	18%	0.03	0.04	0.06	0.15						21%	0.03	0.05	0.07	0.13					
	As	0%	6.72	13.89	38.79	81.22						0%	4.35	6.89	14.2	55.2					
	Pb	0%	0.64	0.84	1.14	1.7											2.6%	0.43	0.7	1.04	2.36
	Tl	0%	0.18	0.22	0.26	0.35											11.35%	0.08	0.11	0.16	0.26
**Phthalate substitute**	OH-DINCH											0.19%	0.98	2.13	4.66	14.7	4.91%	0.29	0.7	6.81	19.82
	oxo-DINCH											1.55%	0.39	0.93	2.03	7.16	13.5%	0.11	0.35	1.19	11.87
	cx-MINCH											0.19%	0.49	1.02	2.11	7.8	3.68%	0.26	0.43	1.22	8.21
**Phthalates**	MEHP	2.4%	1.75	2.53	4.33	8.42						13.0%	0.71	1.22	2.04	4.19	3.68%	2.47	4.09	6.63	14.9
	5OH-MEHP	0%	6.67	10.08	13.75	38.04						0%	5.87	8.98	13.94	28.8	0%	11.54	18.44	26.26	56.6
	5oxo-MEHP	0%	4.39	7.18	9.69	22.98						0%	4.08	6.42	10.49	21.6	0.61%	7.74	11.45	17.03	35.71
	5cx-MEPP											0%	6.1	9.92	16.9	35.8	0%	12.82	18.88	28.15	54.27
	MBzP	0%	3.79	7.1	11.97	22.59						0.39%	1.45	2.38	4.75	17.5	1.23%	3.16	5.09	8.98	28.53
	MnBP	0%	23.36	34	51.35	91.75						0%	12.04	18.18	28.67	54.8	0.61%	9.63	14.74	22.23	41.53
	OH-MnBP											0.78%	1.25	2.12	3.49	7.27	2.45%	1.08	1.71	2.44	5.04
	MiBP	0%	43.16	60	94.4	288.5						0%	13.54	21.36	33.58	87.2	0%	16.33	23.71	34.19	72.73
	OH-MiBP											0%	4.7	7.52	12.17	30.2	0%	6.5	9.17	14.19	25.01
	MEP	0%	17.76	40.38	82.98	203.57						0%	10.96	17.76	32.05	113	0%	87.08	189.47	345.21	1307.09
	OH-MiNP											0%	3.35	5.27	8.73	24.6	1.84%	2.03	3.45	5.91	23.17
	oxo-MiNP											0%	1.39	2.17	3.66	9.65	3.07%	1.19	2.08	3.62	14.93
	cx-MiNP											0%	2.88	4.55	7.5	19.5	0.61%	3.85	6.11	9.91	45.64
	OH-MiDP											0.78%	0.75	1.19	2.06	5.9	1.84%	1.23	1.76	2.83	5.11
	oxo-MiDP											10.5%	0.29	0.54	0.89	2.56	10.4%	0.42	0.62	0.96	1.82
	cx-MiDP											2.14%	0.41	0.7	1.19	3.62	0.61%	1.05	1.43	2.27	4.87
	MMP											1.55%	3.21	5.07	10.44	36.0	4.91%	2.01	2.69	4.2	10.56
**PAHs**	1-OH-Pyr	1.6%	0.11	0.15	0.24	0.49	0%	0.06	0.10	0.13	0.26	1.36%	0.06	0.09	0.14	0.29					
	4-OH-Phe						20%	0.02	0.05	0.12	1.5	0.39%	0.02	0.04	0.08	0.26					
	1-OH-Phe						5.5%	0.08	0.17	0.34	0.70	0%	0.08	0.12	0.2	0.46					
	2-OH-Flu						0%	0.21	0.36	0.56	1.0	10.5%	0.23	0.43	0.69	2.19					
	2-OH-Nap						0%	3.0	5.2	7.1	21	0.19%	1.86	3.15	5.89	15.9					
	1-OH-Nap						0%	1.0	1.7	3.3	6.2	3.5%	0.36	0.68	1.41	4.88					
**Bisphenols**	BPA	2.4%	0.9	1.29	2.3	4.61	0%					3.69%	1.03	1.6	2.88	6.91					
**PFAS**	PFNA						0%	0.23	0.3	0.36	0.49						0.61%	0.7	0.95	1.39	2.14
	PFDA						0%	0.11	0.12	0.17	0.25						11.0%	0.26	0.37	0.53	0.84

**Figure A1:**
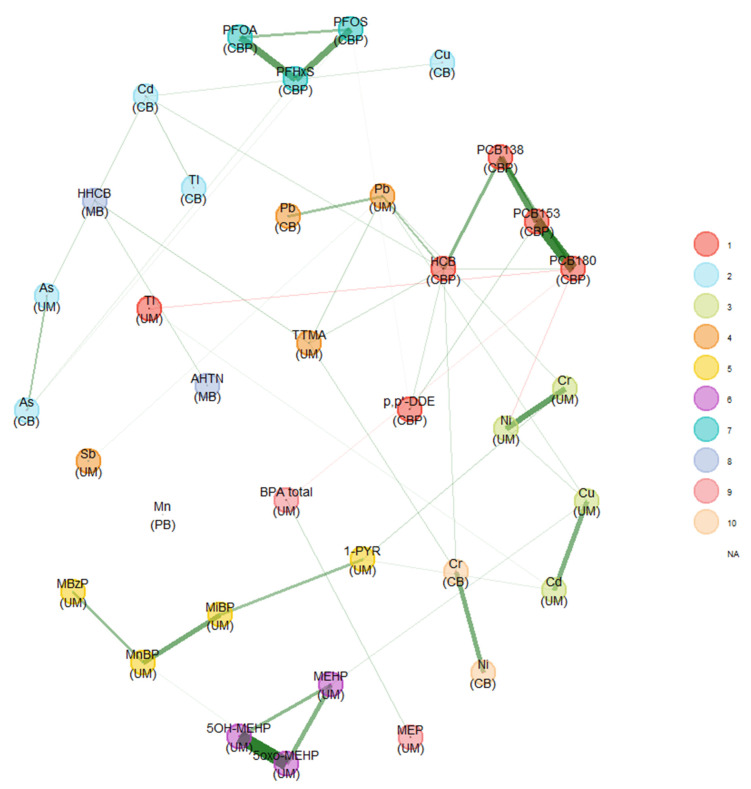
Weighted network for 3xG using the graphical lasso method. Data were corrected for age, smoking, and BMI. Urinary markers were standardised for creatinine; lipid soluble blood markers were standardised for lipids. Matrices in which biomarkers are measured appear between brackets. Green lines represent a positive dependency between nodes (biomarkers), while red lines represent a negative dependency.

**Figure A2:**
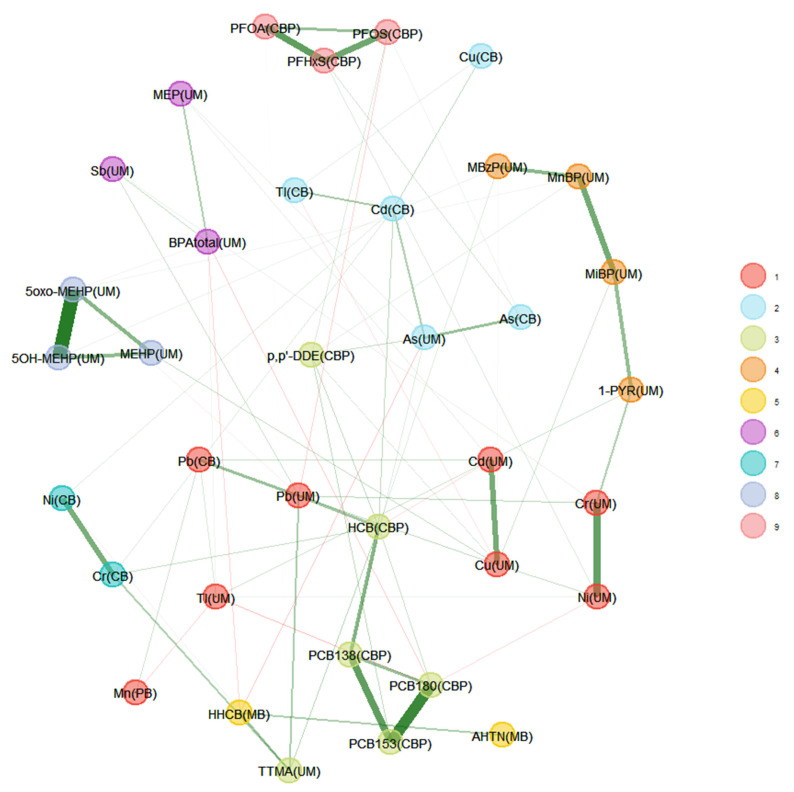
Weighted network for 3xG using the graphical lasso method with bootstrap of 80 iterations. Data were corrected for age, smoking, and BMI. Urinary markers were standardised for creatinine; lipid soluble blood markers were standardised for lipids. Matrices in which biomarkers are measured appear between brackets. Green lines represent a positive dependency between nodes (biomarkers), while red lines represent a negative dependency.

**Figure A3:**
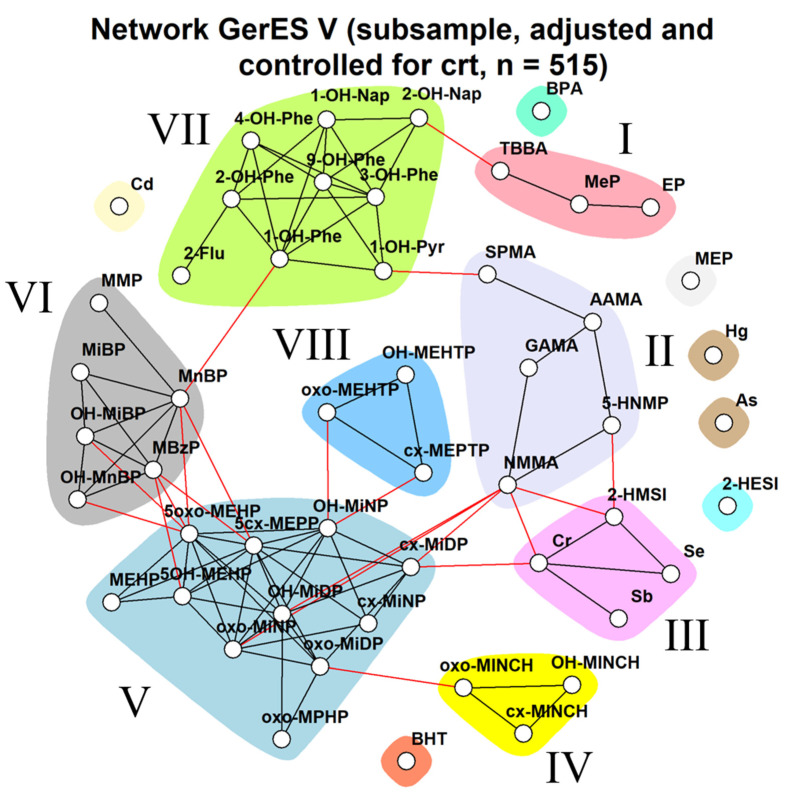
Network of GerES V subset, using creatinine-standardised and creatinine-adjusted data. Black lines indicate dependency between nodes (biomarkers) within a community; red lines indicate dependency between nodes in different communities.

**Figure A4:**
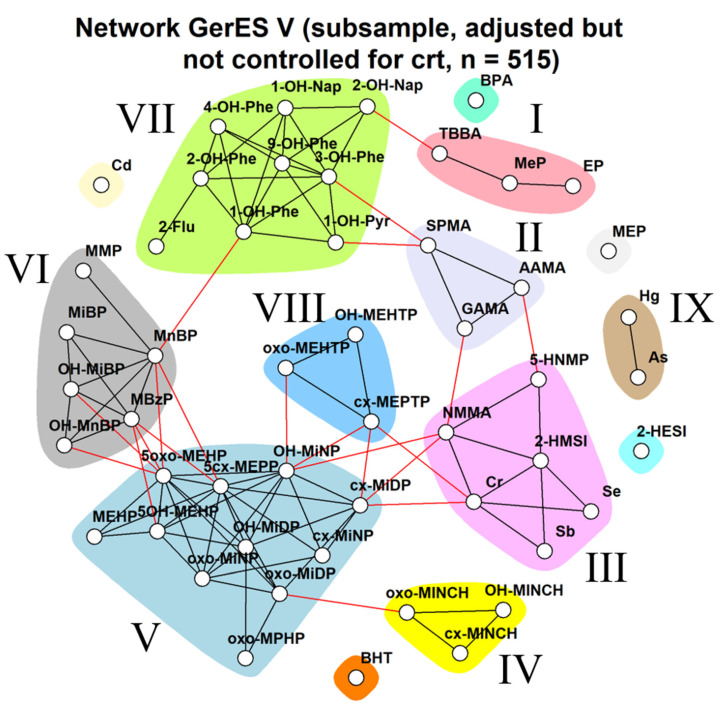
Network of GerES V subset, using creatinine-standardised data. Black lines indicate dependency between nodes (biomarkers) within a community; red lines indicate dependency between nodes in different communities.

**Figure A5:**
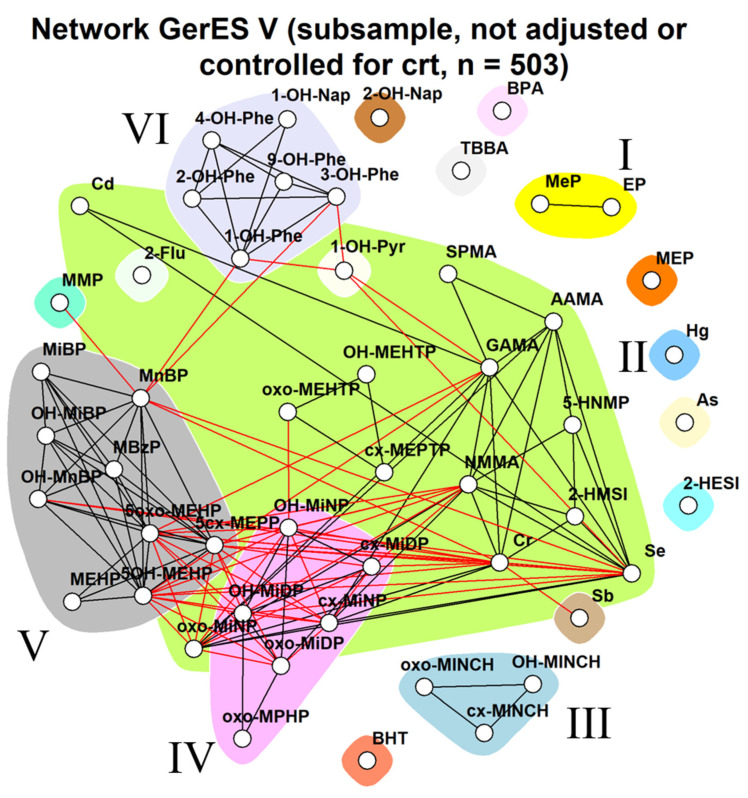
Network of GerES V subset, without application of any correction for creatinine. Black lines indicate dependency between nodes (biomarkers) within a community; red lines indicate dependency between nodes in different communities.

## Data Availability

Summary data are listed in Table 1 and Tables S1–S4. As the individual data are considered pseudonimised data, those cannot be made public according to the European General Data Protection Regulation (GDPR; [37]). Raw data from the CELSPAC—FIREexpo study are available in anonymised form upon request and upon the approval of the steering committee. Aggregated data from the BIOAMBIENT study can be found at IPCHEM (https://ipchem.jrc.ec.europa.eu/, accessed on 27 January 2023).

## Supplementary Materials

The following supporting information can be downloaded at: https://www.mdpi.com/article/10.3390/toxics11030204/s1. Table S1: Descriptive statistics for the biomarkers included in the network analysis for the 3xG study; Table S2: Descriptive statistics for biomarkers included in the network analysis for the CELSPAC—FIREexpo study; Table S3: Descriptive statistics for biomarkers included in the network analysis for the GerEs V study; Table S4: Descriptive statistics for biomarkers included in the network analysis for the BIOAMBIENT.ES study.

## Appendix A

### Comparison of the Two Unweighted and Weighted Network Estimation Approaches

Two methods to visualise weighted networks were explored with the R Package EGAnet (v1.2.3 [21]). For the first method, the EGA() function was applied to the correlation matrix of the data. This function estimates the number of dimensions of the correlation matrix using graphical lasso with extended Bayesian information criterion to select optimal regularisation parameters. Figure A1 shows the resulting weighted network from this method.

The second method uses the function bootEGA() from the EGAnet R package which estimates the number of dimensions of n bootstraps using the empirical (partial) correlation matrix (parametric) or resampling from the empirical dataset (non-parametric). It also estimates a typical median network structure, which is formed by the median or mean pairwise (partial) correlations over the n bootstraps. Here, a parametric bootstrap (1000 iterations) was used to estimate the median network structure, which was then plotted as the final result (shown in Figure A2).

Networks obtained using the two different methods maintain the communities constituted by PFASs, PCBS with HCB and p,p’-DDE, DEHP metabolites, and DiBP metabolites with 1-PYR. The network of MEP and BPA total is conserved; however, Sb is included in the bootstrap network, while in the GLASSO network, it constitutes its own community. The composition of other, less strong communities seems to vary slightly between the two methods; nevertheless, the overall relationships do not seem to differ heavily between the two approaches.

### Impact of Different Approaches to Correcting Biomarker Levels against Creatinine Levels

The impact of different approaches for standardisation of creatinine (or lack thereof) in the network analyses was studied in the GerES V sample (Figure A3, Figure A4 and Figure A5) with networks as described above (using a parametric bootstrap of 1000 iterations). We distinguished between the following terms when taking into account dilution. ‘Standardisation’ means that each individual’s raw concentration for the biomarkers studied is divided by its individual dilution level (e.g., creatinine). ‘Adjustment’ for dilution reflects that the dilution was included as a control variable into multivariate regression (see also [38]). Finally, ‘correction’ is used as the general term of taking into account dilution levels as standardisation, adjustment, or the combination of both. To illustrate the effect of correction of urinary dilution with creatinine, Figure A3 shows the resulting communities when standardising raw concentrations for creatinine and adjusting for creatinine in multivariate analyses (recommended by HBM4EU). A total of eight communities containing three or more substances was observed. The communities are grouped into DINCH metabolites (yellow), PAHs (green), parabens and TBBA (salmon), acrylamide and SPMA (plum), DEHTP metabolites (blue), aprotic solvent HNMP, NMMA, and acrylamide (plum), selenium, chromium, antimony, and aprotic solvent HMSI (lavender), and two communities of phthalate metabolites. Among the phthalate communities, MMP co-occurs together with BBzP, and DnBP and DiBP metabolites (grey), and DEHP metabolites co-occur with DPHP, and DiNP and DiDP metabolites (blue). Several substances were not part of any community, such as some elements (mercury, arsenic, and cadmium), BPA, and the phthalate MEP.

The differences between the network obtained using creatinine-standardised and creatinine-adjusted data (Figure A3) versus using only creatinine-standardised data (Figure A4) are limited. The major differences include an additional community (comprising mercury and arsenic, IX in Figure A4), and a different split of the community that consists of acrylamide, aprotic solvents, selenium, chromium, antimony, NMMA, and SPMA into two different communities (II and III). However, not correcting for creatinine in any form results in considerably different communities. As can be seen in Figure A5, three heavily inter-related communities (II, IV, V) mask the detailed communities detected in the network when standardising and adjusting for creatinine, possibly due to a similar degree of dilution being reflected in stronger correlations. These findings indicate that it is important to correct for creatinine when aiming at analysing mixtures and at least to standardise the biomarker concentrations for this parameter; nevertheless, this needs to be confirmed by further studies. In conclusion, not correcting for dilution effects may create spurious results. The two methods for correction for dilution effects showed little difference.
