# Supplementary material for: Identification of Real-Life Mixtures Using Human Biomonitoring Data: A Proof of Concept Study
Source: Toxics. 2023 Feb 22;11(3):204. doi: 10.3390/toxics11030204 (PMC10058482; doi:10.3390/toxics11030204)
Supplement: Supplementary file 1 [file toxics-11-00204-s001.zip › toxics-2164501-supplementary.pdf]

toxics

MDPI

5Supplementary Materials to:

Identification of real-life mixtures using human biomonitoring data: a proof of concept study

Laura Rodriguez Martin [1], Ilse Ottenbros [2,3], Nina Vogel[4], Marike Kolossa-Gehring[4], Phillipp Schmidt[4], Katarína Řiháčková[5] , Miguel Juliá Molina[6] , Elena Varea-Jiménez[6], Eva Govarts[1] , Susana Pedraza-Diaz[6], Erik Lebret[2,3], Jelle Vlaanderen[2], Mirjam Luijten [7,*]

[1] Health, Flemish Institute for Technological Research (VITO), Mol, Belgium; laura.rodriguezmartin@vito.be; eva.govarts@vito.be
[2] Institute for Risk Assessment Sciences (IRAS), Utrecht University, Utrecht, The Netherlands; i.b.ottenbros@uu.nl; j.j.vlaanderen@uu.nl; lebret.ivori-consult@xs4all.nl
[3] Center for Sustainability, Environment and Health, National Institute for Public Health and the Environment, Bilthoven (RIVM), The Netherlands; Ilse.Ottenbros@rivm.nl; lebret.ivori-consult@xs4all.nl
[4] German Environment Agency (UBA), Berlin, Germany; Nina.Vogel@uba.de; marike.kolossa@uba.de; Phillipp.Schmidt@uba.de
[5] RECETOX, Faculty of Science, Masaryk University, Brno, Czech Republic; katarina.rihackova@recetox.muni.cz
[6] National Centre for Environmental Health, Instituto de Salud Carlos III, Majadahonda, Spain ; mjuliam@isciii.es; evareaj@isciii.es; spedraza@isciii.es
[7] Centre for Health Protection, National Institute for Public Health and the Environment (RIVM), Bilthoven, The Netherlands; Mirjam.Luijten@rivm.nl

* Correspondence: Mirjam.Luijten@rivm.nl; Tel.: +31 6 2549 7106

Supplementary Table S1: Descriptive statistics for biomarkers included in the network analysis for 3xG study. Urinary markers expressed as µg/g creatinine, blood values as µg/L.

Substance group	Substance	Biomarker		% < LOQ	P25	P50	P75	P95
Elements	Cadmium	Cd	Mothers, morning urine (µg/L)	0%	0.15	0.21	0.36	0.65
	Cadmium	Cd	Newborns, cord blood (µg/L)	8%	0.02	0.03	0.04	0.05
	Chromium	Cr	Mothers, morning urine (µg/L)	1.6%	0.24	0.39	0.66	1.4
	Chromium	Cr	Newborns, cord blood (µg/L)	18%	0.14	0.24	0.57	1.3
	Antimony	Sb	Mothers, morning urine (µg/L)	18%	0.02	0.04	0.06	0.09
	Nickel	Ni	Mothers, morning urine (µg/L)	0%	1.3	1.7	2.6	4.7
	Nickel	Ni	Newborns, cord blood (µg/L)	37%	<LOD	0.12	0.2	1.2
	Copper	Cu	Mothers, morning urine (µg/L)	0%	9.3	12	17	29
	Copper	Cu	Newborns, cord blood (µg/L)	0%	540	576	626	698
	Arsenic	As	Mothers, morning urine (µg/L)	0%	0.8	1.2	2.1	4.5
	Arsenic	As	Newborns, cord blood (µg/L)	0%	0.5	0.89	1.6	3.3
	Lead	Pb	Mothers, morning urine (µg/L)	0%	0.51	0.76	1	1.7
	Lead	Pb	Newborns, cord blood (µg/L)	0%	5	6	7	10
	Manganese	Mn	Newborns, cord blood (µg/L)	0%	28	35	44	67
	Thallium	Tl	Mothers, morning urine (µg/L)	0%	0.11	0.18	0.26	0.36
	Thallium	Tl	Newborns, cord blood (µg/L)	0%	0.01	0.02	0.02	0.04
Phthalates	DEHP	MEHP	Mothers, morning urine (µg/L)	2.4%	1.3	1.9	3.8	8.5
		OH-MEHP	Mothers, morning urine (µg/L)	0%	5.3	8.2	13	34
		oxo-MEHP	Mothers, morning urine (µg/L)	0%	3.9	5.7	9.3	20
	BBzP	MBzP	Mothers, morning urine (µg/L)	0%	3	5.8	11	26
	DnBP	MnBP	Mothers, morning urine (µg/L)	0%	18	28	45	119
	DiBP	MiBP	Mothers, morning urine (µg/L)	0%	34	46	79	276
	DEP	MEP	Mothers, morning urine (µg/L)	0%	12	34	77	189
Bisphenols	Bisphenol A	BPA	Mothers, morning urine (µg/L)	2.4%	0.8	1.2	2.1	4.5
PCBs	PCB 138	PCB 138	Newborns, cord blood plasma (µg/L)	6.4%	0.02	0.03	0.03	0.05

Substance group	Substance	Biomarker		% < LOQ	P25	P50	P75	P95
	PCB 153	PCB 153	Newborns, cord blood plasma (µg/L)	1.6%	0.03	0.04	0.05	0.09
	PCB 180	PCB 180	Newborns, cord blood plasma (µg/L)	5.6%	0.02	0.02	0.04	0.07
Persistent organic pollutants	Dichlorodiphenyldi-chloroethylene	DDE	Newborns, cord blood plasma (µg/L)	0%	0.09	0.15	0.26	0.53
	Hexachlorobenzene	HCB	Newborns, cord blood plasma (µg/L)	24.8%	0.01	0.01	0.02	0.03
PFASs	PFOA	PFOA	Newborns, cord blood plasma (µg/L)	0%	0.77	1.1	1.4	2.1
	PFHxS	PFHxS	Newborns, cord blood plasma (µg/L)	20.8%	0.21	0.34	0.46	0.76
	PFOS	PFOS	Newborns, cord blood plasma (µg/L)	0%	1.1	1.6	2.2	3.6
Musks	Galaxolide	HHCB	Mothers, blood (µg/L)	0%	230	282	359	543
	Tonalide	AHTN	Mothers, blood (µg/L)	0.8%	50	63	81	186

Supplementary Table S2: Descriptive statistics for biomarkers included in the network analysis for the CELSPAC – FIREexpo study. Urinary markers expressed as μg/g creatinine, blood values as μg/L.

Study population	Substance group	Biomarker	% <LOQ	LOQ	P10	P25	P50	P75	P90
**Firefighters**	**Blood serum (ng/ml)**								
	PFASs	PFOA	0	0.07	0.66	0.92	1.2	1.5	1.9
		PFNA	0	0.004	0.23	0.29	0.40	0.54	0.63
		PFDA	0	0.004	0.10	0.14	0.19	0.25	0.30
		PFUnDA	12	0.012	<LOQ	0.04	0.05	0.07	0.10
		PFBS	27	0.04	<LOQ	<LOQ	0.15	0.18	0.25
		PFHxS	0	0.004	0.3	0.38	0.49	0.67	0.76
		PFHpS	3.9	0.005	0.04	0.06	0.08	0.10	0.14
		PFOS	0	0.03	1.7	2.4	3.2	4.8	6.4
	**Morning urine (μg/g CRT)**								
	PAHs	1-NAPH	0	0.006	0.52	0.99	1.6	2.6	3.6
		2-NAPH	0	0.006	1.4	2.9	4.2	6.2	9.5
		2-FLUO	0	0.006	0.14	0.19	0.26	0.32	0.48
		3-FLUO	5.8	0.006	0.02	0.04	0.06	0.11	0.16
		1-PHEN	38	0.006	<LOQ	<LOQ	0.02	0.04	0.10
		∑(2-PHEN+ 3-PHEN)	0	0.006	0.07	0.10	0.14	0.20	0.30
		4-PHEN	1.9	0.006	0.04	0.21	0.32	0.50	0.59
		1-PYR	0	0.006	0.04	0.05	0.07	0.10	0.14
**Control group**	**Blood serum (ng/ml)**								
	PFASs	PFPeA	16	0.036	<LOQ	0.18	0.22	0.26	0.31
		PFHxA	7.3	0.04	0.05	0.07	0.08	0.10	0.11
		PFOA	1.8	0.07	0.49	0.69	0.90	1.1	1.4
		PFNA	0	0.004	0.18	0.23	0.30	0.36	0.41
		PFDA	0	0.004	0.08	0.11	0.12	0.17	0.23

		PFUnDA	3.6	0.012	0.03	0.05	0.07	0.10	0.11
		PFHxS	0	0.004	0.27	0.33	0.43	0.52	0.65
		PFHpS	36	0.005	<LOQ	<LOQ	0.04	0.07	0.09
		PFOS	0	0.03	1.1	1.7	2.2	2.7	3.5
	**Morning urine (µg/g creatinine)**								
	PAHs	1-NAPH	0	0.006	0.34	0.58	0.93	1.3	2.3
		2-NAPH	0	0.006	1.3	1.7	2.8	4.1	5.3
		2-FLUO	0	0.006	0.09	0.13	0.18	0.25	0.31
		3-FLUO	3.6	0.006	0.01	0.02	0.03	0.05	0.07
		1-PHEN	5.5	0.006	0.02	0.05	0.09	0.12	0.20
		∑(2-PHEN+ 3-PHEN)	0	0.006	0.06	0.09	0.12	0.20	0.28
		4-PHEN	20	0.006	<LOQ	0.01	0.02	0.04	0.35
		1-PYR	0	0.006	0.02	0.03	0.04	0.06	0.09

Supplementary Table S3: Descriptive statistics for biomarkers included in the network analysis for the GerEs V study. Urinary markers expressed as µg/g creatinine, blood values as µg/L.

Substance group	Substance	Biomarker	N < LOQ	% < LOQ	LOQ	P05	P10	P25	P50	P75	P90	P95	GM
Elements	Cadmium	Cd	134	26.02 %	0.05	< LOQ	< LOQ	< LOQ	0.06	0.09	0.12	0.15	0.06
	Chromium	Cr	40	7.77 %	0.2	< LOQ	0.2	0.26	0.34	0.49	0.62	0.77	0.36
	Mercury	Hg	26	5.05 %	0.02	< LOQ	0.02	0.04	0.06	0.1	0.19	0.26	0.06
	Antimony	Sb	108	20.97 %	0.04	< LOQ	< LOQ	0.03	0.05	0.07	0.1	0.13	0.05
	Selenium	Se	0	0 %	0.5	15.09	16.81	21.17	27.8	37.95	47.93	57.08	28.34
	Arsenic	As	0	0 %	0.1	2.45	2.93	4.35	6.89	14.17	30.5	55.21	8.42
Aprotic solvents		HNMP	0	0 %	2.5	17.78	22.73	31.79	48.6	73.76	107.36	152.65	49.71
		HMSI	0	0 %	2	15.6	19.62	26.81	37.15	57.12	84.41	104.4	39.32
		HESI	66	12.82 %	2	< LOQ	< LOQ	2.58	4.68	10.2	39.19	70.35	5.87
Acrylamide		AAMA	0	0 %	1	26.84	32.62	43.56	60.5	84.27	125.31	189.67	63.22
		GAMA	0	0 %	1	5.52	6.79	8.96	12.53	17.66	24.62	28.84	12.74
Phthalate substitutes	DEHTP	OH-MEHTP	171	33.2 %	0.3	< LOQ	< LOQ	< LOQ	0.41	1.13	2.59	4.23	0.48
		oxo-MEHTP	103	20 %	0.2	< LOQ	< LOQ	0.19	0.46	1.06	2.28	3.83	0.47
		cx-MEPTP	0	0 %	0.2	1.1	1.51	2.85	6.22	15.45	36.64	54.09	6.76
	DINCH	cx-MINCH	1	0.19 %	0.05	0.21	0.29	0.49	1.02	2.11	4.91	7.8	1.08
		OH-MINCH	1	0.19 %	0.05	0.41	0.54	0.98	2.13	4.66	9.48	14.72	2.22
		oxo-MINCH	8	1.55 %	0.05	0.15	0.21	0.39	0.93	2.03	4.6	7.16	0.94
Phthalates	DEHP	MEHP	67	13.01 %	0.5	< LOQ	< LOQ	0.71	1.22	2.04	3.35	4.19	1.2
		5OH-MEHP	0	0 %	0.2	3.12	3.94	5.87	8.98	13.94	21.69	28.84	9.25
		5oxo-MEHP	0	0 %	0.2	1.96	2.52	4.08	6.42	10.49	15.89	21.63	6.47
		5cx-MEPP	0	0 %	0.2	3.45	4.03	6.1	9.92	16.9	26.26	35.81	10.17
	BBzP	MBzP	2	0.39 %	0.2	0.69	0.9	1.45	2.38	4.75	10.36	17.46	2.77
	DnBP	MnBP	0	0 %	1	6.05	7.81	12.04	18.18	28.67	40.34	54.79	18.39
		OH-MnBP	4	0.78 %	0.25	0.6	0.78	1.25	2.12	3.49	5.24	7.27	2.11
	DiBP	MiBP	0	0 %	1	7.29	9.04	13.54	21.36	33.58	58.2	87.22	22.34

Substance group	Substance	Biomarker	N < LOQ	% < LOQ	LOQ	P05	P10	P25	P50	P75	P90	P95	GM
		OH-MiBP	0	0 %	0.25	2.28	3.02	4.7	7.52	12.17	21.02	30.15	7.78
	DEP	MEP	0	0 %	0.5	5.09	6.83	10.96	17.76	32.05	65.95	113.45	19.75
	DiNP	OH-MiNP	0	0 %	0.2	1.88	2.28	3.35	5.27	8.73	15.12	24.62	5.76
		oxo-MiNP	0	0 %	0.2	0.73	0.92	1.39	2.17	3.66	6.35	9.65	2.36
		cx-MiNP	0	0 %	0.2	1.52	1.82	2.88	4.55	7.5	12.48	19.47	4.87
	DiDP	OH-MiDP	4	0.78 %	0.2	0.37	0.47	0.75	1.19	2.06	3.54	5.9	1.28
		oxo-MiDP	54	10.49 %	0.2	< LOQ	< LOQ	0.29	0.54	0.89	1.55	2.56	0.54
		cx-MiDP	11	2.14 %	0.2	0.24	0.3	0.41	0.7	1.19	2.2	3.62	0.76
	DPHP	oxo-MPHP	184	35.73 %	0.25	< LOQ	< LOQ	< LOQ	0.27	0.54	1.01	1.57	0.29
	DMP	MMP	8	1.55 %	1	1.49	1.93	3.21	5.07	10.44	21.45	36	6.02
PAHs		1-OH-Nap	18	3.5 %	0.05	0.11	0.19	0.36	0.68	1.41	3.42	4.88	0.7
		2-OH-Nap	1	0.19 %	0.05	0.92	1.15	1.86	3.15	5.89	11.06	15.89	3.38
		2-OH-Flu	54	10.49 %	0.05	< LOQ	< LOQ	0.23	0.43	0.69	1.27	2.19	0.36
		1-OH-Phe	0	0 %	0.005	0.04	0.05	0.08	0.12	0.2	0.34	0.46	0.13
		2-OH-Phe	4	0.78 %	0.005	0.03	0.03	0.05	0.07	0.11	0.18	0.28	0.08
		3-OH-Phe	0	0 %	0.005	0.05	0.05	0.08	0.11	0.18	0.3	0.4	0.12
		4-OH-Phe	2	0.39 %	0.001	0.01	0.01	0.02	0.04	0.08	0.18	0.26	0.04
		9-OH-Phe	14	2.72 %	0.005	0.01	0.02	0.03	0.05	0.09	0.19	0.28	0.05
		1-OH-Pyr	7	1.36 %	0.01	0.03	0.04	0.06	0.09	0.14	0.22	0.29	0.09
Parabens	Methylparaben	MeP	13	2.52 %	0.5	0.8	1.04	1.9	4.37	19.61	122.99	321.93	7.02
	Ethylparaben	EP	164	31.84 %	0.5	< LOQ	< LOQ	< LOQ	0.62	1.42	4.38	10.39	0.72
Bisphenols	Bisphenol A	BPA	19	3.69 %	0.5	0.52	0.67	1.03	1.6	2.88	4.8	6.91	1.77
Other	Lysmeral	TBBA	0	0 %	0.2	2.12	2.87	4.49	8.16	15.45	24.19	35.56	8.49
	CIT/MIT	NMMA	0	0 %	0.5	2.48	2.99	3.92	5.31	7.53	10.24	12.2	5.47
	Butylhydroxytoluol	BHT	1	0.19 %	0.2	0.58	0.73	1.23	1.98	3.45	6.22	9.52	2.1
	Benzene	SPMA	12	2.33 %	0.02	0.02	0.03	0.05	0.08	0.14	0.26	0.44	0.09

Supplementary Table S4: Descriptive statistics for biomarkers included in the network analysis for the BIOAMBIENT.ES study. Urinary markers expressed as µg/g creatinine, blood values as µg/L.

Substance group	Substance	Biomarker	N < LOQ	% < LOQ	LOQ	P05	P10	P25	P50	P75	P90	P95	GM
Metals	Mercury	Hg	1	0.68	0.10	0.21	0.31	0.56	0.99	1.58	2.3	2.75	0.88
	Cadmium	Cg	4	2.45	0.05	0.05	0.07	0.12	0.2	0.38	0.59	0.72	0.2
	Lead	Pb	4	2.6	0.10	0.19	0.29	0.43	0.7	1.04	1.67	2.36	0.69
	Thallium	Tl	16	11.35	0.05	0.05	0.06	0.08	0.11	0.16	0.21	0.26	0.11
	Cobalt	Co	2	1.46	0.05	0.2	0.23	0.36	0.57	0.84	1.28	2.2	0.58
Phthalates	DMP	MMP	8	4.91	1	1.12	1.46	2.01	2.69	4.2	7.08	10.56	3.14
	DEP	MEP	0	0	0.5	25.81	39.64	87.08	189.47	345.2 10	802.21	1307.09	189.2 4
	BBzP	MBzP	2	1.23	0.2	1.77	2.27	3.16	5.09	8.98	16.24	28.53	5.69
	DiBP	MiBP	0	0	1	8.37	10.89	16.33	23.71	34.19	51.71	72.73	24.04
		OH-MiBP	0	0	0.25	3.71	4.77	6.5	9.17	14.19	19.14	25.01	9.58
	DnBP	MnBP	1	0.61	1	4.76	6.04	9.63	14.74	22.23	33.4	41.53	14.54
		OH-MnBP	4	2.45	0.25	0.56	0.72	1.08	1.71	2.44	3.52	5.04	1.65
		MCPP	22	13.5	0.50	0.42	0.51	0.71	0.95	1.52	2.2	2.9	1.07
	DEHP	MEHP	6	3.68	0.50	1.08	1.43	2.47	4.09	6.63	11.32	14.9	3.97
		OH-MEHP	0	0	0.20	6.24	7.89	11.54	18.44	26.26	39.22	56.6	17.75
		oxo-MEHP	1	0.61	0.20	3.63	4.86	7.74	11.45	17.03	27.82	35.71	11.56
		cx-MEPP	0	0	0.20	7.24	8.29	12.82	18.88	28.15	43.43	54.27	19.19
	DiNP	OH-MiNP	3	1.84	0.20	0.99	1.28	2.03	3.45	5.91	10.97	23.17	3.62
		oxo-MiNP	5	3.07	0.20	0.50	0.71	1.19	2.08	3.62	6.71	14.93	2.2
		cx-MiNP	1	0.61	0.20	0.63	0.76	1.05	1.43	2.27	3.61	4.87	1.59
	DiDP	OH-MiDP	3	1.84	0.20	0.7	0.9	1.23	1.76	2.83	4.09	5.11	1.84
		oxo-MIDP	17	10.43	0.20	0.25	0.29	0.42	0.62	0.95	1.49	1.82	0.64
		cx-MIDP	1	0.61	0.20	0.63	0.76	1.05	1.43	2.27	3.61	4.87	1.59
DINCH	DINCH	OH-DINCH	8	4.91	0.05	0.1	0.15	0.29	0.7	1.9	6.81	19.82	0.85

Substance group	Substance	Biomarker	N < LOQ	% < LOQ	LOQ	P05	P10	P25	P50	P75	P90	P95	GM
		cx-MINCH	6	3.68	0.05	0.09	0.14	0.26	0.43	1.22	4.3	8.21	0.64
		oxo-DINCH	22	13.5	0.05	0.03	0.04	0.11	0.35	1.19	4.6	11.87	0.4
PFAS	PFHxS	PFHXS	34	20.86	0.34	0.24	0.24	0.39	0.68	1.13	1.99	2.39	0.70
	PFOA	PFOA	0	0	0.16	0.81	0.96	1.39	2.03	2.94	3.92	5.09	1.98
	PFOS	PFOS	2	1.23	0.33	2.48	3.53	5.30	8.09	11.02	15.18	17.11	7.25
	PFNA	PFNA	1	0.61	0.16	0.48	0.59	0.70	0.95	1.39	1.74	2.14	0.98
	PFDA	PFDA	18	11.04	0.2	0.14	0.14	0.26	0.37	0.53	0.75	0.84	0.37
